# Deciphering the tumor immune microenvironment from a multidimensional omics perspective: insight into next-generation CAR-T cell immunotherapy and beyond

**DOI:** 10.1186/s12943-024-02047-2

**Published:** 2024-06-26

**Authors:** Zhaokai Zhou, Jiahui Wang, Jiaojiao Wang, Shuai Yang, Ruizhi Wang, Ge Zhang, Zhengrui Li, Run Shi, Zhan Wang, Qiong Lu

**Affiliations:** 1 Department of Pharmacy, The Second Xiangya Hospital, Central South University, Changsha, Hunan 410011 China; 2 Department of Urology, The First Affiliated Hospital of Zhengzhou University, Zhengzhou, Henan 450052 China; 3 Department of Nephrology, Union Medical College Hospital, Chinese Academy of Medical Sciences, PekingBeijing, 100730 China; 4 Department of Cardiology, The First Affiliated Hospital of Zhengzhou University, Zhengzhou, Henan 450052 China; 5 Department of Oral and Maxillofacial-Head and Neck Oncology, Shanghai Ninth People’s Hospital, Shanghai Jiao Tong University School of Medicine, Shanghai, China; 6 Department of Oncology, The First Affiliated Hospital of Nanjing Medical University, Nanjing, China

**Keywords:** Tumor immune microenvironment, Chimeric antigen receptor T cell, Multi-omics, Immunotherapy, Solid tumors, Machine learning

## Abstract

Tumor immune microenvironment (TIME) consists of intra-tumor immunological components and plays a significant role in tumor initiation, progression, metastasis, and response to therapy. Chimeric antigen receptor (CAR)-T cell immunotherapy has revolutionized the cancer treatment paradigm. Although CAR-T cell immunotherapy has emerged as a successful treatment for hematologic malignancies, it remains a conundrum for solid tumors. The heterogeneity of TIME is responsible for poor outcomes in CAR-T cell immunotherapy against solid tumors. The advancement of highly sophisticated technology enhances our exploration in TIME from a multi-omics perspective. In the era of machine learning, multi-omics studies could reveal the characteristics of TIME and its immune resistance mechanism. Therefore, the clinical efficacy of CAR-T cell immunotherapy in solid tumors could be further improved with strategies that target unfavorable conditions in TIME. Herein, this review seeks to investigate the factors influencing TIME formation and propose strategies for improving the effectiveness of CAR-T cell immunotherapy through a multi-omics perspective, with the ultimate goal of developing personalized therapeutic approaches.

## Introduction

Tumor immune microenvironment (TIME), comprising the immunological components and their interactions in tumor microenvironment (TME) niche, has been known for the critical function of shaping tumor development in both the dynamic temporal and spatial dimensions and sensitivity to treatment [1–3]. Miscellaneous immune cells in TIME could regulate tumor-antagonizing activities and are closely correlated with clinical outcomes in cancer [4, 5]. Furthermore, it has been ascertained that the spectrum of tertiary lymphoid structures (TLSs), aggregates of immune cells with a composition comparable to lymph nodes in non-lymphoid tissues, could be indicators of cancer prognosis, progression, and response to immunotherapy [6]. Over the past decade, countless researchers have been working on characterizing TIME, which laid the groundwork for gaining an unparalleled understanding of immune cell composition, function, and spatial distribution within TIME [7, 8]. To date, with the development of cutting-edge technologies such as high-resolution single-cell RNA sequencing (scRNA-seq), flow cytometry, and molecular imaging, the research on TIME has delved into cellular subpopulations and spatial localization. These technological platforms have contributed to the flourishing of multi-omics analysis, heralded as the cornerstone of individualized precision medicine, which further unveiled inter-cellular crosstalk and pivotal mechanisms in TIME [9, 10].

**Fig. 1:**
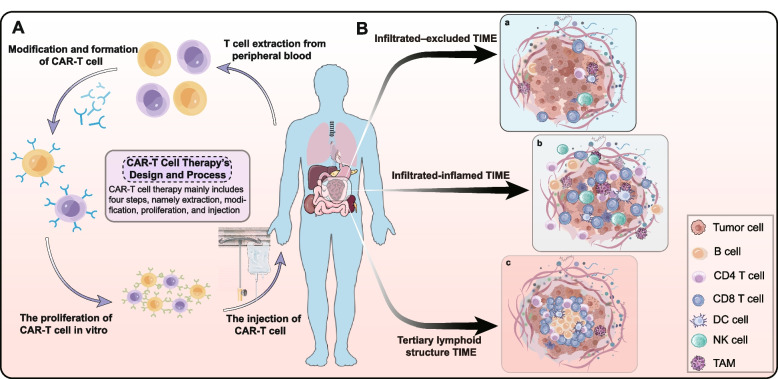
The design of CAR-T cell therapy and a simplified basis for classifying TIME. **A** CAR-T cell therapy’s design and process. The process of CAR-T cell therapy mainly includes four steps: firstly, T cells are extracted from peripheral blood, and then CAR-T cells are created by modification. These CAR-T cells are expanded in vitro and finally injected back into the patient to complete the whole treatment. **B** Three TIME types are classified on the composition of immune infiltrate. In general, TIME can be divided into three types, including infiltrated-excluded (I-E) TIME, infiltrated-inflamed (I-I) TIME, and tertiary lymphoid structure (TLS) TIME. In different tumor ecosystems, the types of TIME serve as innovative biomarkers for cancer treatment/prognosis

Adoptive T cell therapy, especially chimeric antigen receptor (CAR)-T cell immunotherapy, has exhibited exciting successes in hematological malignancies [11]. The treatment is generally extracted from peripheral blood T cells of tumor patients, cultured and modified in vitro, and then equipped with special molecules to recognize and attack specific cancer cells. The modified T cells with specifically targeted tumor-killing capability are injected back into the patient to battle the tumor (Fig. 1A) [12]. Frustratingly, the scope of utility and potential life-threatening toxicity of CAR-T cell in solid tumors remains a Gordian knot. With further in-depth decoding of tumor immunological profiles, it has been demonstrated that manipulating the sophisticated TIME could bring novel insights to CAR-T cell immunotherapy [13]. Multi-omics studies deeply revealed the properties and immune resistance mechanisms of TIME, which will provide valuable insights into CAR-T cell immunotherapy [14].

A more thorough dissection of the immune infiltrates profile in cancer lesions is essential to developing efficient CAR-T cell immunotherapy. More significantly, multi-omics offers unique opportunities to dissect the sophistication and heterogeneity of TIME, further augmenting sufficient signaling strength and durability of CAR-T cells. Herein, we first summarized the established classification and spatial architecture of TIME. Secondly, we discussed the elements influencing the formation of TIME, especially from host–tumor genome perspective. Ultimately, optimizing CAR-T cell strategies from multidimensional omics data and machine learning (ML) was discussed to provide precision medicine-based decision-making.

## Classification and spatial architecture of the TIME

### Classification of the TIME

A simplified basis for classifying TIME has been formulated based on the composition of immune infiltrate and characteristics of the inflammatory response (Fig. 1B) [2]. The infiltrated–excluded (I–E) TIME subtype was widely populated with immune cells in the stroma but relatively lacked cytotoxic lymphocytes (CTLs) in tumor parenchyma, whose CTLs localized along the perimeter of the tumor. It was hypothesized to be ‘cold’ or poorly immunogenic [15]. CTLs of I-E TIMEs showed low expression of the activation markers, such as *IFNG* and *GZMB (GRZB)*, which were characteristic of immune ignorance, namely the inability to elicit high cytotoxicity against tumor cells [16]. In comparison, infiltrated-inflamed (I-I) TIMEs were defined by high infiltration with various immune cells such as CTLs, B cells, and T cells. I-I TIMEs were considered to be immunologically ‘hot’ tumors, which allowed more robust immune responses for immune checkpoint inhibitors (ICIs). TLS TIMEs, a subclass of I-I TIMEs, had histological evidence of containing TLSs. TLSs are frequently [17, 18] but not always linked to a favorable prognosis [19]. The classification of TIME is quite seminal to comprehending the impact of immunological composition and condition on overall survival, predicting and guiding immunotherapeutic responsiveness, and revealing novel therapeutic targets.

### Spatial architecture of the TIME

A more elaborated spatial characterization of components depending on higher-resolution techniques improves our comprehension of TIME. The spatial structure was described comprehensively based on the location of immune cells and immunomodulators and distance between cells within TIME [20, 21].

#### Distribution of immune cells and immune checkpoints in TIME

**Fig. 2:**
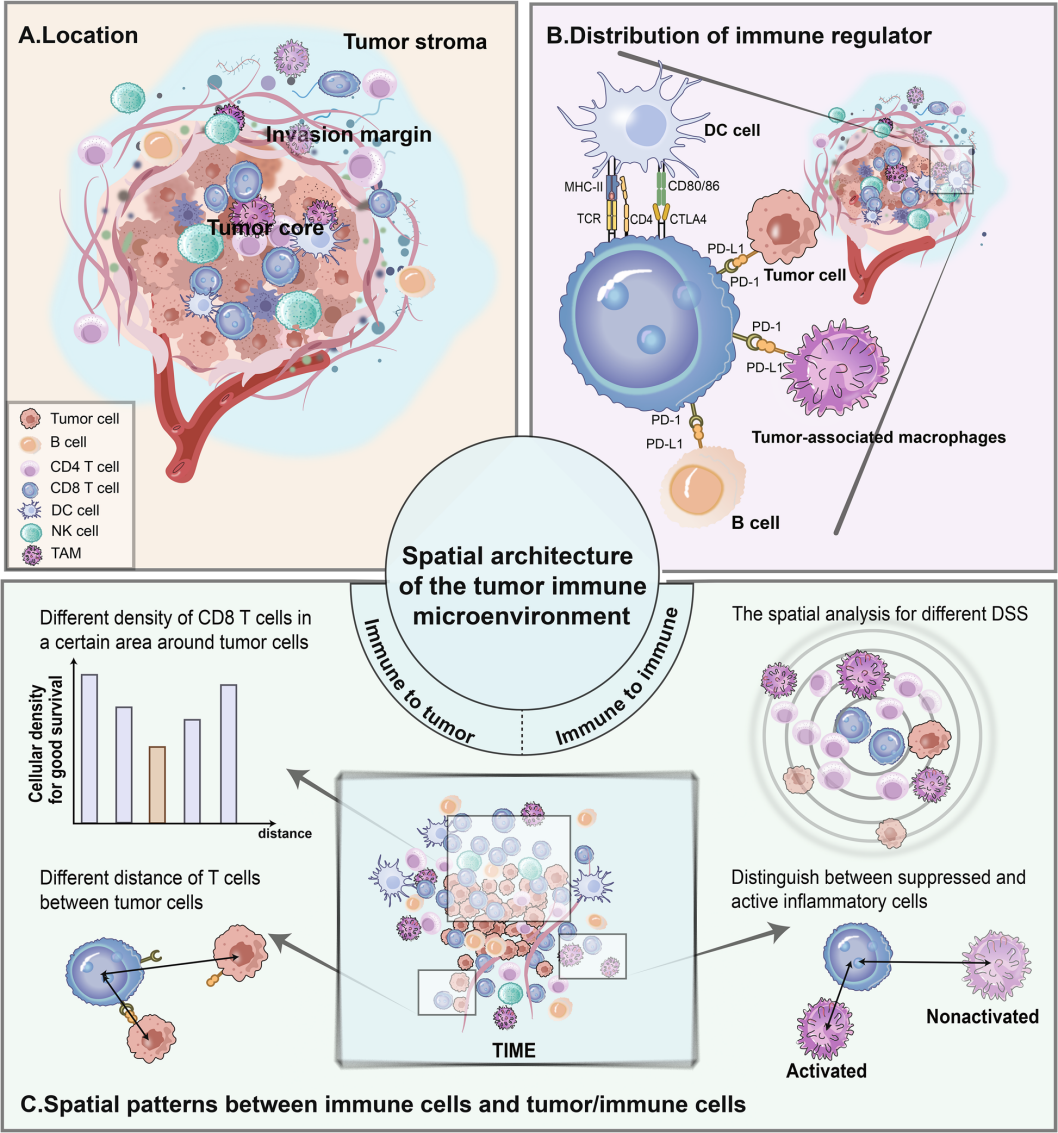
Spatial architecture of the tumor immune microenvironment (TIME). **A** The location of immune cells. Categorizing immune cells based on the compartments in which they reside in tumor tissue: the tumor core, also known as the tumor nest or tumor cluster, houses the majority of tumor cells; the invasive margin, transition zone between the tumor core and the tumor stroma, comprises abundant infiltrating immune cells, such as T cell, B cell, NK cell and DC cell; the tumor stroma, located around the tumor core, contains rich stromal components, providing nutrients for the tumor. **B** Distribution of immune checkpoints. The spatial distribution of immunomodulatory molecules in TIME shows regularity. PD-1 is mainly expressed on the surface of CD8 + T cells, whereas its ligand PD-L1 is expressed on the surface of various cells, such as B cells, tumor cells, and tumor-associated macrophages. The regulatory relationship between T cells and DC cells is mediated by TCR, CD4, and CTLA4 on T cells and MHC-II and CD80/86 on DC cells. **C** Spatial patterns between immune cells and tumor/immune cells. For the spatial relationship between immune cells and tumor cells, the distance between them and immune cell density can be analyzed. Distance between immune cells and tumor cells may affect the lethality of immune cells to tumors as well as the editing effect of tumor cells on immune cells. In addition to directly affecting the interaction between tumor cells and immune cells, CD8 + T cells have different densities in specific regions at different distances from tumor cells. The size of its density is likely to influence the exertion of its effects. For the spatial pattern preceding immune cells, analysis is mainly performed from distance as well. The distance between immune cells reflects the interactions within the immune cell population. Quantification and spatial analysis between immune cells can be used to distinguish suppressed nonfunctional immune cells from functionally active immune cells, and further information on disease-specific survival can be derived

Localization of immune cells within compartments indicated specific relationships with tumor cells and other immune components (Fig. 2A) [22]. The tumor compartment is comprised of three parts: tumor stroma (supplying nutrients to the tumor), tumor core (main concentration of tumor cells), and infiltrative margin (transition zone between tumor cells and normal cells), suggesting a physical or functional border within different regions [23–25]. Immune cells dwell in separate compartments, which exhibit alternative phenotypic states and characteristics [26]. Immune cells in tumor stoma are closely correlated with stromal remodeling and angiogenesis, potentially exerting a profound influence on tumor development, infiltration, and metastasis, while those within tumor nests or tumor clusters possessed enhanced communication and interaction with tumor cells [27, 28]. Tumor margin is the main battlefield in fighting against cancer and the density of tumor margin-infiltrating immune cells is considerably abundant compared to other sites [29]. Analysis of integrating spatial resolution with laser capture microdissection gene expression profiles to stratify TIME validated that CD8 + T cells differ significantly in terms of immunosuppressive molecules and functional biomarkers in these three regions [22]. Remarkably, owing to diverse tissue origins of the tumors, as well as the high flux and variability of immune cells, there is also heterogeneity in the spatial distribution of immune cells within different regions.

ICIs have yielded prominent clinical efficacy in patients with several tumors. Detecting the spatial location of immune targets/regulators is the next step in scaling up the application in clinical practice (Fig. 2B). Spatially resolved and multiparametric single-cell analysis unveiled immune checkpoints such as programmed death-1 (PD-1), lymphocyte activation gene-3 (LAG-3), and T cell immunoglobulin and mucin-domain containing-3 (TIM-3) that have distinct tissue/cell distribution, functional implications, and genomic correlates [30]. The hypothesised Interaction Distribution (HID) method, an automated multiplex approach, was constructed to decode the complexity of immune cell interactions. Spatial proximity between T cells and PD-1 ligand (PD-L1) expressing cells could be quantified through HID, which was considered to reflect immune escape and generate prognostic information [31]. As immunomodulators interact through ligand-receptor binding, the distance between the two immune targets could influence tumor immune activity. Quantitative assessment densities and geographic interactions between PD-1 + cells and PD-L1 + cells were demonstrated as a nexus with the anti-PD-1 therapy response [32]. Likewise, quantitative spatial profiling of key immunosuppressive mechanisms in TIME could help select which subset of melanoma patients are more amenable to PD-1 monotherapy, allowing for individualized dosing [33]. Nevertheless, the applicability of spatial architecture of these immune targets should be further explored in clinical research.

#### Distance between immune cells and tumor/immune cells in TIME

Distance between immune cells and tumor cells may directly impinge on the killing effect of immune cells on tumors or, conversely, the editing of immune cells by tumor cells (Fig. 2C) [34]. Evolving technologies with higher flux, dimensionality, and resolution could detect the distinct density patterns of immune cell localization in correlation to malignant tissue [35–37]. Based on cell densities, cell-to-cell distances, and spatial heterogeneity, the immune contexture biomarkers of tumor data-driven identification could provide mathematical definitions of different feature classes. It has been demonstrated that distances of tumor cells and immune cells were potentially prognostic and predictive markers [38]. The spatial dimension between lymphocytes and tumor buds has been proven to forecast the tumor prognostic progression, such as colorectal cancer (CRC) and melanoma [39, 40]. Employing multiplex immunofluorescence analysis, Tuba et al*.* have demonstrated that the spatial arrangement of immune and tumor cells could affect the intensity of immune response to anti-PD-1-based therapies [41]. In periampullary and pancreatic adenocarcinoma, a detailed in situ description of lymphocyte infiltration patterns showed that the proximity of CD8α + cells to tumor cells was associated with overall survival [42]. It was found that high T cell counts at the direct tumor border were strongly coupled with improved overall survival of CRC liver metastases patients. Interestingly, at a distance of 20 to 30 µm from the tumor, the decrease in T cells was found to be significantly associated with improved survival [25].

Distance between immune cells and immune cells potentially captures the interactions prevalent in immune cell populations and facilitates investigators to better fathom all immune cells as a coherent whole (Fig. 2C) [43]. Quantification and spatial analysis of tumor-infiltrating inflammatory cells have the potential to distinguish between suppressed non-functionally and functionally active inflammatory cells [44]. CTLs were closer to activated macrophages than to non-activated ones, which is associated with disease-specific survival (DSS) [40]. Specifically, CTL distance to macrophages was linked to poor DSS, whereas the distance to tumor cells was correlated inversely with DSS. The presence of lymphocytes with CD4 + T-helper capacities in the nearest vicinity to CD8α + cells was associated with prolonged overall survival [42].

## Multi-omics characteristics shape TIME profiles

**Fig. 3:**
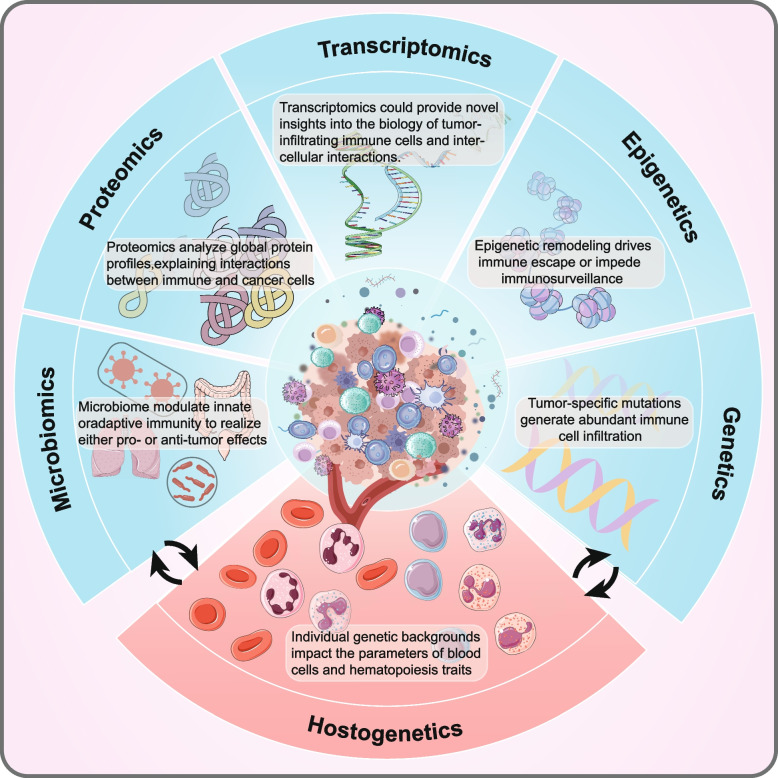
Multi-omics characteristics shape TIME profiles. Although the precise composition of TIME is currently not well understood, it is certain that tumor and host characteristics are thought to subtly promote specific tumor immune ecosystems. With the advancement of high-throughput technology, germline genetic data/hostogenetics on the innate immune profile of tumors have been shown. Germline genetics profoundly impacts the composition and functional localization of a variety of immune cells in TIME, such as T cells, NK cells, and B cells. In comparison, tumor omics landscapes are discussed. The extent and nature of immune infiltration may be influenced by the overall mutational landscape of neoplastic cells, serving as a direct indicator of tumor immunogenicity. As a relative part of genetics, gene mutations can drive changes in epigenetic modifications and epigenetic remodeling critically shapes cancer development by altering gene expression, promoting immune evasion, or hindering immunosurveillance. Transcriptomics offers new insights into the biology of immune cells infiltrating tumors and their intercellular interactions in TIME. Changes in the transcriptome of tumor cells impact the expression of immune-related genes, triggering pathways associated with tumor immune escape, and then influencing the infiltration and activity of immune cells. Emerging proteomics studies protein composition and alterations, complementing genomics and transcriptomics in revealing key proteins, molecular mechanisms, and pathways in TIME. It offers precise insights into interactions between immune and cancer cells. The microbiome could either promote pro- or anti-tumor effects, impacting responses to chemotherapy and immunotherapy. Specifically, the intratumoral microbiome alters T cell repertoires and microbial metabolites, influencing immune homeostasis, anti-tumor surveillance, and tumorigenesis

Although precise constituents dictating the composition of TIME remain incompletely understood, it could be hypothesized that there is a complex interactivity between tumor genotype/phenotype and immune characteristics. Tumor and host characteristics such as oncogenic mutations, germline genetics, and microbiome, were believed to subtly promote specific tumor immune ecosystems, thereby modulating the prevalence of distinct inflammatory states and influencing immune response degree **(**Fig. 3**)** [45, 46]. Among these elements, it is conceivable that the battle of “tumor genome-to-human genome” or host–tumor crosstalk represents a cornerstone in predisposing patients to developing I-I, I-E, or TLS TIME. Due to the heterogeneity of TIME and host, even individuals harboring the same type of tumor could exhibit unique immunological profiles. Moreover, a diverse array of cytokines, chemokines, and immune molecules collectively orchestrate an inflammatory or non-inflammatory milieu [46, 47].

### Hostogenetics

Hostogenetics, namely the genetics of the host, could partially elucidate how germline variants delicately orchestrate TIME profile. Research on autoimmune and infectious disease phenotypes exhibited that the entirety of germline genetic variation was involved in regulating immune responses, such as susceptibilities to chronic inflammation [48–50]. Although germline genetic data on the inherent immune profile of tumors are still scarce, the mystery of hostogenetics is gradually being unwrapped with advancements in high-throughput technologies. How variants relate to immune cells or how environmental and genetic factors affect innate and adaptive immune responses have been explored to decode TIME. A large-scale blood cell trait variation data including 563,085 participants was integrated to identify the genetic architecture of hematopoiesis, assess correlated genetic models of blood cell phenotypes, and characterize relevant hematopoietic cell states subject to regulated genetic variation and gene networks. Results manifested that individual genetic backgrounds could impact the relative parameters of blood cells and hematopoiesis traits [51]. Cohort studies probing the effects of variation on immune cell traits further highlighted immune cells are surrounded by complex genetic regulation [52]. Etienne and colleagues performing flow cytometry of blood leukocytes and genome-wide DNA genotyping found that innate cells were more susceptible to the control of genetic variation than adaptive cells [53]. These genetic association studies will establish a resource generating innovative hypotheses about TIME and hostogenetics.

To explore tumor-immune interactions, the implications of frequent and rare germline variants on well-defined immune traits were researched in cancer recipients. It was observed that natural killer (NK) cell and T cell subset infiltration and interferon signaling possessed high heritability and germline genetics profoundly impact the composition and functional localization of TIME. Mechanistically, common variants such as IFIH1, STING1, and TMEM108 were implicated in divergent interferon pathways, variants localized to RBL1 affiliated with T cell subpopulation proficiency, and variants in BRCA1 and Wnt-β-catenin could exert the agency of immunomodulators [54]. The expression of immune genes could be shaped by hereditary genetic variants to control immune cell abundance within TIME [55]. Besides, some gene signatures from tumor transcriptome data proved to predict patient prognosis, and such gene signatures intensity could be ascertained by germline genetic variation [56, 57].

### The genetics and epigenetics landscape of the tumor

The degree and phenotype of immune infiltration could be modulated by overarching the tumor mutational landscape, which directly reflects tumor immunogenicity. Mutant peptides bound to major histocompatibility complex (MHC) class I/II molecules could generate anti-tumor activity. Higher tumor mutational burden could augment the likelihood of generating immunogenic mutations that serve as prime targets for T cell-mediated attack [58–60]. These mutations could remodel the cellular phenotypes and spatial interactions of TIME. In BRCA1/2mut ovarian cancer, a proliferating tumor-cell subpopulation has been reported to be associated with enhanced spatial tumor-immune interactions by CD8 + and CD4 + T cells [61]. Early mutations, which manifest at the inception of oncogenesis and are ubiquitously present across malignant cell populations, could potentially evoke a more robust T-cell-mediated anti-tumor response compared to branch mutations that arise subsequently and only impact a subset of neoplastic cells [62]. Genomic sequencing data suggested that neoantigens arising as a consequence of tumor-specific mutations possessed robust immunogenicity to activate abundant immune cell infiltration [63]. MHC class I-associated neoantigens, recurrently mutated genes, and genetic amplifications were closely correlated with cytolytic activity [64].

Epigenetic remodeling plays a critical role in tumor development/progression by altering gene expression to drive immune escape or impede immunosurveillance [65]. Mutations in certain genes could lead to altered epigenetic modifications, thereby silencing tumor suppressor genes or activating oncogenes to reshape tumor immune ecosystem and immunogenicity. Cytokine secretion was modulated by epigenetic alterations at gene and chromatin scales during the development of diverse T lymphocyte subsets [66]. Tumor progression tended to be accompanied by elevated DNA methylation and mRNA levels than adjacent non-tumorous tissues, which could affect immune factor expression profiles to modulate TIME and immune response against tumors [67, 68]. Tumor epigenetic modifications were directly associated with the expression of key cytokines such as interleukin 1B (IL1B), IL6, and IL8 to be involved in inflammatory response [68]. Methylation and demethylation for PD-L1 gene promoter could result in immune checkpoint constitutive or dampened expression. TGF-β1 decreased DNMT1 (DNA methylation enzyme) content and led to PD-L1 promoter demethylation whereas tumor necrosis factor-α (TNF-α) induced NF-κB pathway to promote expression of demethylated PD-L1 promoter [69].

### Transcriptome

Transcriptomic profiling was used in diverse areas of cancer research, such as tumor diagnosis, assessing tumor aggressiveness and prognosis, and biomarker discovery. Transcriptomics could provide novel insights into the biology of tumor-infiltrating immune cells and inter-cellular interactions in TIME [70]. Nevertheless, understanding how tumor intrinsic transcriptomic alterations contribute to the immune cell content of TIME is still not clear because of the enormous excess of cancer cells in the tumor mass. Recently, by investigating immune checkpoints transcriptome profile, researchers uncovered immunoreceptor tyrosine-based inhibitory motif (ITIM) domains (T cell immunoreceptor with Ig and ITIM domains, TIGIT) expression on behalf of aggressiveness tumor could impact not only T cells but also other immune cells, thereby manipulating anti-tumor immune [71]. Najwa et al*.* concluded that cytotoxic T lymphocyte-associated antigen 4 (CTLA-4) and PD-1/PD-L1 expression could be interfered with, either directly or indirectly, by microRNAs repressing mRNA expression at a post-transcriptional level. MicroRNAs such as miR-145, miR-3609, and miR-140 in TIME could serve as novel treatment targets to mediate the immunosuppressive ecosystem [72]. Currently, RNA sequencing has evolved from bulk sequencing to single-molecular, single-cell, and spatial transcriptome sequencing, which has been utilized to explore TIME [73, 74]. To depict the immune spectrum in TIME, Yu and colleagues using immunohistochemistry and genomics pipeline scRNA-seq disclosed that tumor expression of CD47 is associated with the level of CD68 pan-macrophages. Intriguingly, CD47 blockade significantly remodeled intratumoral lymphocyte and macrophage compartments by increasing pro-inflammatory macrophages and reducing anti-inflammatory macrophages [75]. Immune-targeted single-cell profiling strategy was designed by using immune-targeted scRNA-seq and a targeted antibody panel for mass cytometry to explore the molecular portrait of immune cell compositions and cell states [76]. Taken together, transcriptomic profiling represents a powerful approach for TIME-oriented research [70, 77].

### Proteomics

Emerging proteomics directly studies the composition and alteration patterns of protein, providing complementary information to genomics and transcriptomics, elucidating the most relevant proteins, molecular mechanisms, and signaling pathways in TIME. Mass spectrometry (MS)-based proteomics have enabled precise and immense protein identification, characterization, and quantification and also served as the comprehensive method for exploring protein interactions, sub-cellular localization, and protein post-translational modifications [78]. Lehtiö and colleagues performed MS-based proteogenomics analysis on non-small cell lung cancer to identify proteomic subtypes based on immune cell composition and specific drive pathways [79]. The same strategy was applied to identify lung squamous cell carcinoma molecular subtypes. Further analysis supported the regulation of metabolic pathways through crosstalk between post-translational modifications including ubiquitination, phosphorylation, and acetylation [80].

Cytokines could bind specific receptors on target cells, triggering a cascade of signaling events that regulate immune responses, cell migration, adhesion, activation, and survival. Phosphoproteomics could uncover the key players in the immune signaling process. Tanzer et al*.* revealed phosphorylation-dependent translocations of numerous proteins stimulated via TNF and the critical role of CDK phosphorylation in the immune response signaling cascade [81]. Proteomic and immunopeptiomic analysis demonstrated the induction of ribosome frameshifting and numerous abnormal trans-frame peptides at cell surface after IFN-γ exposure, indicating that IFN-γ-induced IDO1-mediated tryptophan depletion affects immune recognition by promoting diversification of peptide landscape [82]. Some researchers performed a bottom-up proteomic and lipidomic analysis of extracellular vesicles (EVs) from tumor-associated macrophages (TAMs). Interestingly, TAM-EVs presented proteomic profiles associated with Th1/M1 macrophage polarization, and enhanced inflammatory responses. Furthermore, TAM-EVs possessed bioactive lipids and biosynthetic enzymes altering pro-inflammatory signals in TIME [83].

MS-based single-cell proteomics could analyze global protein profiles in a single cell, explaining cell complexity and diversity, and providing more accurate information about interactions between immune cells and cancer cells. In microsatellite stable CRC, Cytometry by Time-Of-Flight (CyTOF), and RNA-seq analysis showed that chemokines/cytokines recruit immunosuppressive and exhausted T cell subsets, elucidating the specific T cell phenotypes and immunosuppression functional status of CRC ecosystems [84]. In the initial and recurrent glioblastoma, the decreased proportion of TAMs and the increased proportion of exhausted T cells, infiltrating Treg cells, and non-functional NK cells could be identified via CyTOF, providing a comprehensive landscape of intricate immune microenvironment [85]. Overall, proteomics approaches accelerate the understanding of molecular mechanisms, complex cellular networks, and interactions between tumor cells and immune cells.

### Microbiome

There is a complicated cross-reactivity between TIME and microbiome. Accumulating microbiota-immune system evidence suggested that the microbiome could modulate innate and adaptive immunity to realize either pro- or anti-tumor effects and remodel response to chemotherapy and immunotherapy [86, 87]. Tumor microbiome could reshape the tumor immune system by re-editing T cell repertoires and microbial metabolites to influence immune homeostasis, anti-tumor immunosurveillance, and tumorigenesis [88]. Notably, immune microbiome interactions are commonly encountered in respiratory and gastrointestinal tumors [89]. To illustrate, the microbial community in the digestive tract could constitute a component of TIME and facilitate carcinogenesis by causing DNA damage and regulating inflammation [90].

Tumor-microbiome communications could affect immune ecosystem compositions and the relative abundance of immune cells to manipulate immune activity, ultimately influencing patient short-term and/or long-term survival. Intratumoral microbiome has been demonstrated as greatly abundant in tumors than in normal tissues, such as pancreatic cancer [91], and hepatocellular carcinoma [92]. Diversity of Intratumoral microbiota was correlated with immunogenic reprogramming of TIME, comprising qualitative and quantitative alterations in myeloid-derived suppressor cells (MDSCs), NK cells, and M1-polarized macrophages, CD4 + T cells differentiation, and CD8 + T cells activation [93–95]. Microbiota dysregulation could cause gene mutations and activate immunomodulatory factors and signaling to alter the overall composition of immune cells [96–98]. Various epithelial cancers like CRC develop and progress proximally to microbial communities. Sergei et al*.* investigated inflammation mechanisms using the colorectal tumorigenesis mouse model and proposed that MDSCs were likely to be activated by microbial products to produce IL-23 signaling, thereby triggering tumor-elicited inflammation and driving tumor growth/progression [99]. Furthermore, microbial sequencing and reconstitution of germ-free mice have indicated both positive and negative regulatory bacteria likely exist, which either promote or interfere with checkpoint-targeted immunotherapy by regulating PD-1/CTLA-4 expression [100, 101]. Although researchers had a deeper understanding of microbiome in TIME or enhancing anti-tumor immunity, sophisticated molecular mechanisms and cross-talk networks between microbiome and TIME need to be further deciphered.

## Advancing the efficacy of *CAR*-T cell immunotherapy from multi-omics perspectives

The insights derived from the multidimensional omics data of TIME allow for a comprehensive understanding for tumor immunology, which is critical for the development of next-generation CAR-T cell therapies. Specifically, the multi-omics approach enables the identification of novel biomarkers for patient stratification and the optimization of CAR-T cells to target these biomarkers effectively.

### Optimize target antigen choice

Antigen escapes dramatically hamper the efficacy of CAR-T cell therapy. In patients receiving CAR-T cells, a significant proportion of malignant cells have a partial or complete loss of target antigen expression, eventually leading to tumor cell antigen tolerance [102]. Some normal tissues also express target antigens to varying degrees and thus may be mistakenly killed by CAR-T cells, which will put patients at high risk of on-target off-tumor toxicity [103]. Therefore, selecting optimal target antigens with high and specific expression is pivotal to ensuring anti-tumor efficacy and limiting the adverse effects [104, 105]. Pleasantly, although the determination of such target antigens is quite challenging, multi-omics could provide a promising protocol.

Cell surface proteome, namely surfaceome, has suggested a plethora of potential surface targets for treating various malignancies. Leung and colleagues applied quantitative proteomics of N-linked glycoproteins to reveal how the surface and glycoproteome were substantially remodeled in breast epithelial cell lines. Comparing transcriptomic and proteomic data from tumor and normal tissues, various over-expressed cell surface molecules meeting the criteria for CAR-T therapeutic targets were identified [106]. Harnessing RNA sequencing to confirm highly different expressions of Glypican 2 (GPC2) across multiple pediatric brain tumors, researchers designed GPC2-directed CAR-T cells that could safely target malignant tumors with local delivery [107]. Promisingly, the integration of over 500 clinical trial data with transcriptional and proteomics data has facilitated the development of a comprehensive “targeted landscape” for CAR-T immunotherapy (Table 1) [108].

**Table 1:** Neoantigens in clinical trials for adoptive cell transfer therapy

Neoantigen	Cancer type	Biological Intervention	Phase	Enrollment	NCT number	Status	Result
EGFRvIII	Esophagus Cancer|Hepatoma|Glioma|Gastric Cancer	Anti-EGFRvIII CAR T cells	I/II	50	NCT03941626	Recruiting	-
	Glioblastoma Multiforme	Anti-EGFRvIII CAR T cells	I	20	NCT02844062	Unknown	-
	Malignant Glioma|Glioblastoma|Brain Cancer|Gliosarcoma	EGFRvIII CAR PBL	I/II	18	NCT01454596	Completed	Posted
	Recurrent Glioblastoma	EGFRvIII CAR T cells	Early I	22	NCT05802693	Not yet recruiting	-
	Glioblastoma	CART-EGFRvIII T cells	I	7	NCT03726515	Completed	-
	Residual/Reccurent EGFRvIII + Glioma	CART-EGFRvIII T cells	I	11	NCT02209376	Terminated	-
KRAS mut	Pancreatic Cancer|Pancreatic Neoplasms|Pancreatic Ductal Adenocarcinoma|Advanced Cancer	Mutant KRAS G12V-TCR T cells	I/II	30	NCT04146298	Recruiting	-
	Pancreatic Cancer	Mutant KRAS-TCR T Cells	Early I	18	NCT05438667	Recruiting	-
	Solid Tumor	KRAS-EphA-2-CAR-DC	I	10	NCT05631899	Recruiting	-
	Metastatic Colorectal Adenocarcinoma|Metastatic Lung Non-Small Cell Carcinoma|Metastatic Pancreatic Adenocarcinoma	KRAS G12V-TCR T cells	I	24	NCT06043713	Recruiting	-
	Gastrointestinal Cancer|Pancreatic Cancer|Gastric Cancer|Colon Cancer|Rectal Cancer	anti-KRAS G12D TCR PBL	I/II	70	NCT03745326	Recruiting	-
	Pancreatic Cancer|Gastric Cancer|Gastrointestinal Cancer|Colon Cancer|Rectal Cancer	anti-KRAS G12V mTCR PBL	I/II	110	NCT03190941	Recruiting	-
MUC1 mut	Advanced Solid Tumor	Anti-CTLA-4/PD-1 expressing Tn-MUC1 CAR T cells	I/II	40	NCT03179007	Unknown	-
	Advanced Breast Cancer|Breast Neoplasm Malignant	AJMUC1-PD-1 knockout Anti-MUC1-CAR T cells	I/II	15	NCT05812326	Completed	-
	Malignant Glioma of Brain|Colorectal Carcinoma|Gastric Carcinoma	Anti-MUC1 CAR T cells	I/II	20	NCT02617134	Unknown	-
	Advanced Esophageal Cancer	Anti-Tn-MUC1 CAR T cells	I/II	20	NCT03706326	Unknown	-
	Lung Neoplasm Malignant|Non-small Cell Lung Cancer	Anti-MUC1 CAR T cells	I/II	60	NCT03525782	Unknown	-
	Hepatocellular Carcinoma|Non-small Cell Lung Cancer|Pancreatic Carcinoma|Triple-Negative Invasive Breast Carcinoma	Anti-Tn-MUC1 CAR T cells	I/II	20	NCT02587689	Unknown	-
	Intrahepatic Cholangiocarcinoma	Anti-Tn-MUC1 CAR T cells	I/II	9	NCT03633773	Recruiting	-
	Breast Cancer|Ovarian Cancer|Non Small Cell Lung Cancer|Colorectal Cancer|Pancreatic Cancer|Renal Cell Carcinoma|Nasopharyngeal Cancer|Head and Neck Squamous Cell Carcinoma|Gastric Cancer	P-MUC1C-ALLO1 CAR T cells	I	100	NCT05239143	Recruiting	-
TP53 mut	Gynecologic cancer|Colorectal cancer|Pancreatic cancer|Non-small cell lung cancer|Cholangiocarcinoma	TP53 R175H-TCR-T|TP53 R248W-TCR-T|TP53 Y220C-TCR-T cells	I/II	180	NCT05194735	Active, not recruiting	-
	Local Advanced/Metastatic Solid Tumors	TP53-EphA-2-CAR-DC	I	10	NCT05631886	Recruiting	-
	Non-small Cell Lung Cancer|Head and Neck Squamous Cell Carcinoma|Colorectal Carcinoma|Pancreatic Adenocarcinoma|Breast Cancer|Other Solid Tumors	TP53 R175H-TCR T Cells	I	24	NCT05877599	Recruiting	-
HERV	Renalcellcarcinoma	HERV-E TCR T Cells	I	24	NCT03354390	Active, not recruiting	-
HPV	Human Papillomavirus (HPV) 16 + Relapsed/Refractory Cancer	HPV E7-TCR(KITE-439) T cells	I	7	NCT03912831	Terminated	-
	Papillomavirus Infections|Cervical Intraepithelial Neoplasia|Carcinoma In Situ|Vulvar Neoplasms|Vulvar Diseases	HPV E7-TCR T cells	I/II	180	NCT02858310	Recruiting	-
	Vaginal Cancer|Cervical Cancer|Anal Cancer|Penile Cancer|Oropharyngeal Cancer	HPV E6-TCR T cells	I/II	12	NCT02280811	Completed	Posted
	Cervical Cancer|Head and Neck Squamous Cell Carcinoma	HPV E6-TCR T cells	I	20	NCT03578406	Unknown	-
EBV	Nasopharyngeal Carcinoma	EBV-TCR T (YT-E001) cells	II	20	NCT03648697	Unknown	-
	Nasopharyngeal Carcinoma	LMP2 TCR T cells	I	27	NCT03925896	Unknown	-
	Head and Neck Squamous Cell Carcinoma	EBV-TCR T cells	I/II	18	NCT04139057	Recruiting	-
	Nasopharyngeal Carcinoma	EBV-TCR T cells	I/II	20	NCT04509726	Recruiting	-

*mut* Mutation, *MUC1* Mucin 1, *AJMUC1* Aberrantly glycosylated MUC1, *Tn-MUC1(GalNAcα1-O-Ser/Thr)* Glycoform of MUC1, *TP53* Tumor protein p53, *DC* Dentric cell, *PBL* Peripheral blood lymphocyte, *HERV* Human endogenous retroviruses, *LMP2* latent membrane protein 2

**Fig. 4:**
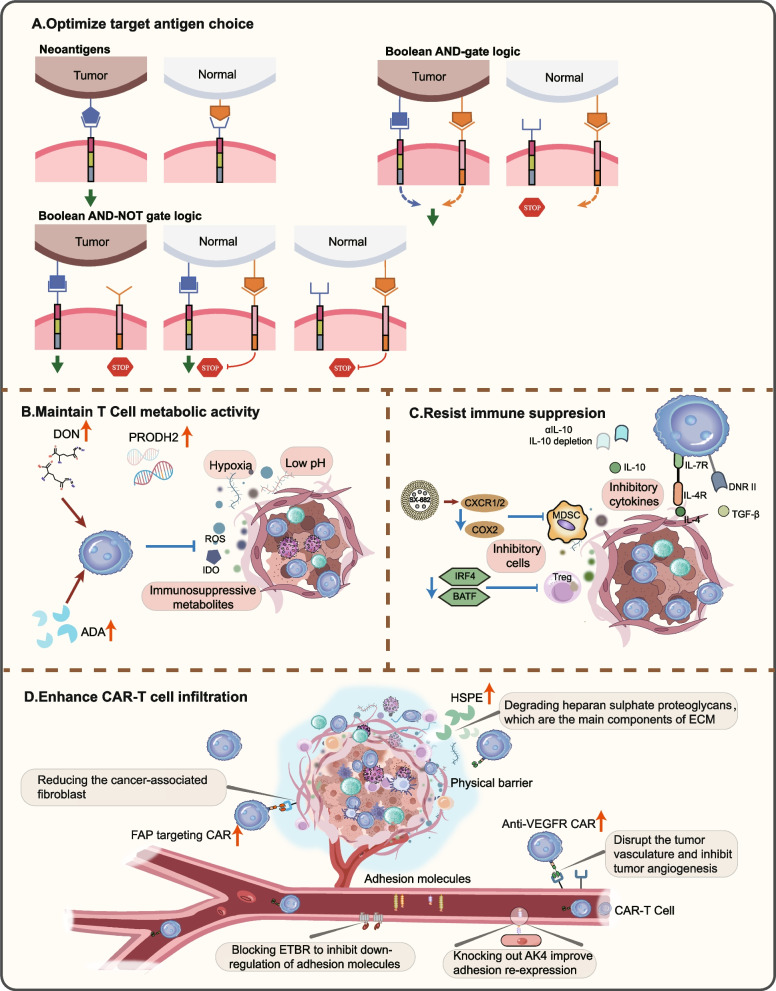
Applications of multi-omics data to overcome barriers of CAR-T cells in the solid tumor microenvironment. **A** Optimize the target antigen choice. Transcriptomics and/or proteomics data from tumor and normal tissue samples can facilitate the discovery of tumor cell-specific neoantigens and optimize the design of chimeric antigen receptor (CAR). Neoantigens are expressed only on the surface of tumor cells and are absent from normal cells. In AND-gate logic, each of the two receptors must bind to the own cognate antigen to elicit a complete T cell signaling response. AND-NOT gate logic refers that only when the tumor associated antigens (TAAs) are present (true) and antigens of normal cells are absent (not true), can T cells be activated. **B** Maintain T cell metabolic activity. Metabolic milieu within the tumor is characterized by hypoxia, low pH, and accumulation of immunosuppressive metabolites. Engineering CAR-T cells to overexpress adenosine deaminase 1 (ADA), which can catabolize adenosine (an adverse metabolites accumulated in TME) into inosine. At the same time, 6-Diazo-5-oxo-l-norleucine (DON) can cause CAR-T cells to retain more T_N_ or T_CM_ subsets and exhibit stronger elimination of burden in vivo. Metabolism reprogramming in CAR-T cells by PRODH2 engineering to improve proline and arginine metabolism can enhance OXPHOS and mitochondrial fitness as well. **C** Resist immunosuppression. CXCR1/2 inhibitors (such as SX-682, an oral small molecule inhibitor of CXCR1/2) or COX-2 pathway blockage can deplete MDSCs, a type of immunosuppressive cells. Tregs are equipped with a strong immunosuppressive ability in TIME, whose immunosuppressive effect can be by suppressed by IRF4 and BATF deletion. Blocking inhibitory cytokines such as IL-4 and TGF-β with specific CAR is also an effective method. **D** Enhance CAR-T cell infiltration. Degrading heparan sulphate proteoglycans (the main components of ECM) by HPSE is a fruitful approach. Cancer-associated fibroblast can be reduced by fibroblast activation protein (FAP)-targeting CAR. Similarly, anti-VEGFR-CAR can be used in disrupting the tumor vasculature and inhibiting tumor angiogenesis. AK4 knockout induces adhesion protein re-expression in EC, inhibits tumor growth, and improves T cell infiltration, while blocking ETBR can inhibit its function on downregulation of adhesion molecules

Neoantigens are personalized antigens produced by specific alterations within tumor cells, such as genomic variation, aberrant mRNA splicing, and dysregulated post-translational modification [109]. Unlike tumor-associated antigens, neoantigens such as epidermal growth factor receptor variant III (EGFRvIII) and Tn glycoform of MUC1 are absent in normal tissues and considered "non-self". The high immunogenicity and tumor specificity of neoantigens make them optimal candidates for targeted immunotherapy (Fig. 4A) [110, 111]. Currently, utilizing genomic and transcriptomic data could screen appropriate candidate antigens, followed by MS to validate. Whole-exome sequencing (WES) and RNA-seq data based on next-generation sequencing (NGS) enable highly sensitive identification of tumor mutated genes, while MS directly detects MHC-present peptides, verifying the expression of mutant mRNA and peptides at the protein level. The combined application of WES and MS has become a powerful weapon for exploring tumor neoantigens for CAR-T immunotherapy [111–113]. Gros et al*.* utilized WES to find neoantigens in gastrointestinal cancer and provided a method to generate specific T cells against identified neoantigens [114]. By employing similar techniques, other researchers constructed EGFRvIII/Anti-Tn-MUC1 CAR-T cells. EGFRvIII/Anti-Tn-MUC1 CAR-T cells demonstrated specific recognition and targeted killing of tumor cells to inhibit tumor growth [115, 116].

Emerging studies have exploited more sophisticated CAR constructs that only function when certain combinatorial CAR-Targets are presented, which could boost engineered cell activation and maximize tumor-targeting specificity of CAR molecules [117]. Specifically, two primary multiple-input receptor combinations: In Boolean AND-gate logic, AND-gate logic CAR requires the presence of two antigens, namely CAR signaling and T cell activation, to activate the CAR. In another design, CARs are activated only when stimulated by tumor antigens and in the absence of antigens normally expressed on healthy cells, thus yielding Boolean AND-NOT gate logic **(**Fig. 4A**)** [103]. Multi-omics data has been used to explore the combinatorial targets of logic-gated CARs [118]. Based on sizeable extensive transcriptomics and proteomics databases from malignant and normal tissues in acute myeloid leukemia, suitable pairs such as CD33 + ADGRE2, CLEC12A + CCR1, CD33 + CD70, and LILRB2 + CLEC12A were found to be capable of enhancing CAR-T effect without increasing extra-tumor toxicity [119]. High-throughput transcriptomics and proteomics allow us to characterize genes encoding cancer cell surface proteins and identify potential combinations of CAR targets [120]. RNA sequencing approaches have been leveraged for a comprehensive in silico screen to recognize features of multiple antigen combinations to provide the predicted antigen pairs and antigen triples **(**Table 2**)**, improving tumor discrimination by CAR-T immunotherapy [121].

**Table 2:** Antigen Pairs Predicted to Improve Tumor Recognition

Cancer type	Pairs	Gate
**Uveal Melanoma**	TRPM1:GPR143	AND gate
	MLANA:GPR143	AND gate
	TSPAN12:CD44	AND-NOT gate
	TRPM1:CLEC2A	AND-NOT gate
	TRPM1:DSC1	AND-NOT gate
	TRPM1:LY6G6C	AND-NOT gate
**Brain Lower Grade Glioma**	TNFRSF9:CACNG7	AND-NOT gate
	BSG:CACNG7	AND gate
	ROM1:CACNG7	AND gate
**Cholangiocarcinoma**	KAAG1:UMOD	AND-NOT gate
	KAAG1:SLC22A11	AND-NOT gate
	KAAG1:SLC34A1	AND-NOT gate
**Colon adenocarcinoma**	GPA33:TMIGD1	AND-NOT gate
	GPA33:MUC17	AND-NOT gate
	GPA33:SI	AND-NOT gate
**Glioblastoma Multiforme**	PCDHGC5:GPR19	AND gate
	NTSR2:GPR19	AND gate
	ERBB2:CACNG7	AND-NOT gate
	DLL3:SYT4	AND-NOT gate
**Kidney renal clear cell carcinoma**	CA12:TREM2	AND gate
**Kidney renal papillary cell carcinoma**	SLC22A2:SLC22A8	AND-NOT gate
	SLC22A2:AQP2	AND-NOT gate
	SLC22A2:SLC12A3	AND-NOT gate
**Liver hepatocellular carcinoma**	GPC3:TM4SF4	AND gate
	TM4SF5:GPC3	AND gate
**Mesothelioma**	TREM2:BDKRB1	AND gate
	UPK1B:UPK2	AND-NOT gate
**Pancreatic adenocarcinoma**	SULF1:OR2I1P	AND gate
**Pheochromocytoma and Paraganglioma**	ZFYVE27:CHRNA3	AND gate
	NRCAM:SLC18A2	AND gate
	CHRNA3:ERBB2	AND-NOT gate
	SLC4A8:CELSR1	AND-NOT gate
	CACNG2:AMER2	AND-NOT gate
	CACNG2:CACNG7	AND-NOT gate
	CACNG2:HRH3	AND-NOT gate
**Prostate adenocarcinoma**	BMPR1B:GPR160	AND gate
**Rectum Adenocarcinoma**	GPR35:LIFR	AND-NOT gate
	TRPM1:CLEC2A	AND-NOT gate
	TRPM1:DSC1	AND-NOT gate
	TRPM1:LY6G6C	AND-NOT gate
**Stomach adenocarcinoma**	MUC13:CLCA1	AND-NOT gate
	MUC13:LYPD8	AND-NOT gate
**Thymoma**	PLEK2:SLCO5A1	AND gate
**Thyroid carcinoma**	DCSTAMP:MS4A15	AND-NOT gate

*TRPM1* Transient receptor potential cation channel subfamily M member 1, *GPR143* G protein-coupled receptor 143, *MLANA* Melan-A, *TSPAN12* Tetraspanin 12, *CLEC2A* C-type lectin domain family 2 member A, *DSC1* Desmocollin 1, *LY6G6C* Lymphocyte antigen 6 family member G6C, *TNFRSF9* TNF receptor superfamily member 9, *CACNG7* Calcium voltage-gated channel auxiliary subunit gamma 7, *BSG* Basigin, *ROM1* Retinal outer segment membrane protein 1, *UMOD* Uromodulin, *KAAG1* Kidney associated DCDC2 antisense RNA 1, *SLC34A1* Solute carrier family 34 member 1, *GPA33* Glycoprotein A33, *TMIGD1* Transmembrane and immunoglobulin domain containing 1, *MUC17* Cell surface associated, *SI* Sucrase-isomaltase, *PCDHGC5* Protocadherin gamma subfamily C, 5, *GPR19* G protein-coupled receptor 19, *NTSR2* Neurotensin receptor 2, *ERBB2* Erb-b2 receptor tyrosine kinase 2, *DLL3* Delta like canonical Notch ligand 3, *SYT4* Synaptotagmin 4, *CA12* Carbonic anhydrase 12, *TREM2* Triggering receptor expressed on myeloid cells 2, *SLC22A2/SLC22A8/SLC12A3* Solute carrier family 22 member 2/8/3, *AQP2* Aquaporin 2, *GPC3* Glypican 3, *TM4SF4/5* Transmembrane 4 L six family member 4/5, *BDKRB1* Bradykinin receptor B1, *UPK1B/2* Uroplakin 1B/2, *SULF1* Sulfatase 1, *OR2I1P* Olfactory receptor family 2 subfamily I member 1 pseudogene, *ZFYVE27* Zinc finger FYVE-type containing 27, *CHRNA3* Cholinergic receptor nicotinic alpha 3 subunit, *NRCAM* Neuronal cell adhesion molecule

### Maintain T cell metabolic activity

Tumor could "starve" effector lymphocytes by creating the suppressive metabolic milieu characterized by hypoxia, low pH, and accumulated immunosuppressive metabolites [122]. The energy requirements of CAR-T cells to maintain effective responses in TIME cannot be met, thus affecting the tumor-control effect [122, 123]. Metabolomics could describe the full physiological state of cells at a certain moment and analyze critical metabolites or intermediates in metabolic disorders. Utilizing gas chromatograph-mass spectrometer (GC–MS), liquid chromatograph-mass spectrometer (LC–MS), and nuclear magnetic resonance (NMR) techniques, researchers could simultaneously identify differences across whole metabolome from a qualitative perspective and characterize significantly different compounds (namely discovery metabolomics), as well as quantify the differential expression of specific metabolites (namely target metabolomics) [124]. Proverbially, sustained antigen exposure could induce metabolic changes in tumor-infiltrating T cells, resulting in an exhaustion phenotype. The metabolic phenotype was characterized by rapid induction of mitochondrial oxidative stress, which restricted T cell ability to engage in oxidative phosphorylation, thereby inhibiting T cell proliferation [125, 126]. Antioxidant therapy thus encourages CAR-T cell self-renewal and enhances anti-tumor capacity [126]. Metabolomics indicated that the transition of T cell differentiation subsets is highly interlinked with metabolic fitness. It was reported that the addition of glutamine antagonist 6-Diazo-5-oxo-l-norleucine (DON) to the culture caused CAR-T cells to retain more T_N_ or T_CM_ subsets and exhibit stronger elimination of burden in vivo. Glutamine inhibition in vitro would be a promising approach to modulate metabolic and differential status to enhance CAR-T therapeutic efficacy [127].

CD28/4-1BB CAR-T cells have shown differential proliferation and persistence in clinical practice, partly attributed to metabolic alterations in engineered CAR-T cells favoring oxidative phosphorylation and aerobic glycolysis, respectively [128]. This offers compelling evidence for the plasticity of T cell metabolic reprogramming, illustrating the feasibility of improving CAR-T cell metabolic fitness in immunosuppressive environments. Adenosine deaminase 1 (ADA) decomposes adenosine into inosine, an indispensable metabolite for T cell functional immune. Altered global gene expression and metabolic signatures were revealed through high-throughput transcriptomics and metabolomics in ADA-engineered CAR-T cells. ADA overexpression significantly enhanced the expansion, tumor infiltration, and tumor control of CAR-T cells in the both preclinical engineered ovarian carcinoma xenograft model and in vivo CRC model [129, 130]. Moreover, increased PRODH2 expression could reshape gene expression and metabolic programs. PRODH2 engineering greatly enhanced CAR-T cell metabolic function and anti-tumor immunity [131]. Overall, multi-omics sheds light on how to counteract the metabolic suppression in TIME at different dimensions and confer superior and durable therapeutic potential to engineered CAR-T cells (Fig. 4B).

### Resist immunosuppression in TIME

Immunosuppression in TIME at multiple mechanistic levels is the stumbling block for the clinical application of CAR-T therapy. Tumor cells could induce preexisting immune cells towards tumor-promoting phenotypes and recruit a range of immunosuppressive cells. Overexpressed immunosuppressive factors such as TGF-β and vascular endothelial growth factor (VEGF) not only prevented cytotoxic T cell infiltration and recruited tumor-promoting cells but also directly impaired T cell function (Fig. 4C) [2, 132]. To fully exert anti-tumor function, CAR-T cells must overcome immunosuppression in TIME. Multi-omics analysis can help us to decipher the composition of the various cell types and cellular states in the TME.

#### Immunosuppressive cells

Based on single-cell transcriptomic data regarding cellular composition of TIME, MDSC characteristics are continuously being exploited. Transcriptomics showed that PTGS2/COX2 and chemokine receptor CXCR2 are among the most up-regulated genes in MDSCs [133, 134]. Oral administration of SX-682, a small molecule inhibitor of CXCR1/2, significantly attenuated MDSCs accumulation in TIME and enhanced NK-cell immunotherapy [135]. Interestingly, SX-682 didn’t exert direct effects, but selectively inhibited CXCR2 + polymorphonuclear (PMN)-MDSC trafficking into tumors, thus enhancing the response to PD-1/PD-L1 axis ICIs and adoptive cell therapy [136]. Burga et al. came to a similar conclusion that CAR-T efficacy was rescued when the tumor received CAR-T in combination with MDSCs depletion [137]. Cyclooxygenase (COX) 2 is a key enzyme converting arachidonic acid to prostaglandins. COX-2 pathway could support directly MDSCs development and CCL2-mediated accumulation while reducing CXCL10-mediated CD8 + T cell infiltration. COX blockade therapy held the potential to suppress tumorigenesis and strengthen T cell anti-tumor immunity [138].

Regulatory T cells (Tregs), especially CD4 + Tregs, are equipped with a strong immunosuppressive ability in TIME. High-dimensional single-cell analysis of T cells identified transcription factor IRF4 that was specifically expressed in a subset of intratumoral CD4 + Tregs with superior suppressive activity. IRF4 + Tregs expressed an army of inhibitory molecules and correlated with T cell exhaustion. Integrative transcriptomic and epigenomics data showed that IRF4, alone or in combination with BATF, was directly responsible for tumor immunosuppression. IRF4 depletion in Tregs resulted in delayed tumor growth and the abundance of IRF4 + Tregs was associated with an inferior prognosis [139]. Furthermore, cooperation of IRF4 and BATF could counteract T cell exhaustion and improve anti-tumor response of CAR-T cells [140]. Utilizing bulk RNA‐seq, immunohistochemistry, and flow cytometry, a remarkable enrichment of Tregs, M2 macrophages, and conventional dendritic cells (cDC2) in hypoxic-high tumor areas was uncovered. Further results showed that Tregs mediated the loss of HLA-DR in the cDC2 subset, which led to immunosuppressive TIME in hypoxic hepatocellular carcinoma, providing a promising thread for CAR construction design [141]. Thus, targeting immune suppressor cells under multi-omics guidance could be a promising strategy to reverse immunosuppression on CAR-T cells in TIME and improve clinical outcomes.

#### Immunosuppressive cytokines

Proteomics, studying ultimate effector that exerts immune function, is a particularly appropriate tool to identify cytokines such as TNF-α, IFN-γ, TGF-β, VEGF, IL-6, and IL-10 [142]. Valid approach to withstand immunosuppression in TIME is engineering T cells to restrict cytokine-induced suppressive signals. Perturb-map, a spatial functional genomics platform combining CRISPR, multiplex imaging, and spatial transcriptomics, could identify genetic determinants of growth, histopathology, and immune composition in TIME*.* Through Perturb-map, Dhainaut and colleagues found that in TGF-βR2 knockout tumors, T cell infiltration was reduced along with upregulated TGF-β and TGF-β-mediated fibroblast activation, indicating that TGF-β receptor loss on tumor cells increased TGF-β relative concentration and its immunosuppressive effect [143]. Knockdown of endogenous TGF-β receptor II (TGF-βR2) in CAR-T cells with CRISPR/Cas9 technology could reduce induced Tregs transformation and prevent CAR-T cell exhaustion (TEx). TGFBR2-engineered CAR-T cells exhibited superior survival and proliferation and generated sustained anti-tumor efficacy in tumor xenograft mouse models [144]. Alternatively, Kloss et al. designed PSMA-specific, TGF-β-insensitive (dnTGF-βRII-T2A-Pbbz) CAR-T cells that exhibited increased proliferation, enhanced cytokine secretion, prolonged persistence, and induced tumor eradication (NCT03089203) [145]. Another strategy targeting immunosuppressive cytokines is engineering T cells with CAR for rewired responses to suppressive soluble ligands. T cells engineered with a CAR responding to TGF-β could convert the suppressive cytokine to a potent stimulant. TGF-β CAR-T cells could protect neighboring immune cells from the immunosuppressive effects of TGF-β, thereby boosting anti-tumor immunity [146]. IL-10 is considered a context-dependent cytokine. IL-10 exhibited carcinogenic function by activating the STAT3 pathway and inducing Tregs production and exerted anti-tumor effects by downregulating angiogenic factors [147]. Combining scRNA-seq, reversed-phase protein arrays, and time-lapse fluorescent microscopy, some researchers revealed the high expression of IL-10 receptor gene in T cells and TAMs and found that IL-10 blockade enhanced MHC-I pathway and macrophage MHC-II-dependent antigen presentation, increasing CD8 + cytotoxic T cell frequency [148]. Neutralizing antibodies against IL-10 (αIL-10) or IL-10 depletion could boost carcinoembryonic antigen (CEA)-specific CAR-T cell activation and CAR-T cell-mediated cytotoxicity [148, 149].

In addition to targeting inhibitory cytokines in TIME, CAR-T cells could be equipped with ability to secrete pro-inflammatory cytokines such as IL-12 and IL18 to remodel suppressive TIME and boost anti-tumor effects. CyTOF analysis demonstrated that IL-18-secreting CAR-T cells could induce the expansion of NK cells, DCs, and endogenous CD8 + T cells and regulate endogenous immune cells phenotype, thereby reversing immunosuppressive TIME and amplifying anti-tumor response [150].

### Enhance CAR-T cell infiltration

TIME poses multiple challenges for CAR-T cells, some of which occur even before encountering tumor cells. Nevertheless, multi-omics era has brought dawn for more efficacious CAR-T cells. Mounting evidence suggests that harnessing multi-omics data to elucidate pivotal factors for overcoming tortuous barriers could reverse deficient CAR-T cell infiltration into TIME. As proof (Fig. 4D): 1) extracellular matrix (ECM) within solid tumor was altered to be more rigid and contractile, creating physical barriers to exclude tumor-infiltrating T cells [151]. The release of specific enzymes like acetaparinase (HPSE, enzyme breaking down ECM-heparan sulfate proteoglycan) is the cornerstone for T cell degradation of ECM [152]. Investigation combined with multi-omics has described the HPSE mRNA downregulation and HPSE protein loss in long-term ex vivo expanded T cells and in ex vivo-engineered and cultured CAR-T cells, causing an impaired ability to degrade ECM. HPSE-expressing CAR-T cells could achieve better tumor infiltration and exert stronger anti-tumor activity, especially in mesenchymal-rich tumors [153]. 2) Aberrant vasculature could hinder CAR-T cell migration to tumor lesions. Expression of adhesion molecules necessary for infiltration such as intercellular cell adhesion molecule (ICAM)-1 and CD34 were down-regulated on endothelial cells (ECs), resulting in inefficient T-cell adhesion [154]. Kinome-wide genetic screening of mesenchymal-like transcriptional activation identified PAK4 as a critical aberrant vascularization regulator. PAK4 knockout induced adhesion protein re-expression in EC, inhibited tumor growth, improved T cell infiltration, and sensitized CAR-T cell immunotherapy [155]. Metabolomic and transcriptomic analyses showed that PHGDH overexpression affected EC glycolysis through a redox-dependent mechanism, leading to EC overgrowth. Endothelial metabolism reprogramming against PHGDH could inhibit aberrant angiogenesis, restore vascular delivery, and thus enhance CAR-T cell infiltration into tumor [156]. 3) Dysregulated chemokines profiles in TIME favor tumor proliferation and metastasis while avoiding anti-tumor inflammatory cell recruitment [157]. ScRNA-seq data demonstrated that STING agonists DMXAA or cGAMP could greatly enhance the tumor control of CAR-T cells, and boost CAR-T cell trafficking and persistence with altered chemokine milieu [158].

### Prevent CAR-T cell exhaustion

Under sustained antigen stimulation, CAR-T cells exhibit a hierarchical loss of effector function, upregulated expression of multiple inhibitory receptors such as PD-1 and LAG3, metabolic dysregulation, and eventual clonal deletion, namely TEx [159, 160]. Transcriptional profiling on exhausted CD8 + T cells has revealed significant alterations in metabolism, cell cycle regulation, and transcription factor expression [161]. TEx Gene expression regulation was closely related to the epigenetic landscape [162, 163]. Genome editing revealed that PD-1 expression was partially regulated by exhaustion-specific enhancers including essential RAR, T-bet, and Sox3 motifs [162]. Advancing transcriptomic and epigenetic techniques have emerged as fruitful methods to elucidate the TEx mechanisms [163].

Single-cell assay for transposase-accessible chromatin using sequencing (scATAC-seq) allows for precise and large-scale deconstruction of complex gene expression programs. Based on ATAC-seq and RNA-seq experiments, Pritykin and colleagues mapped CD8 + T cell differentiation trajectories leading to dysfunction and its underlying transcriptional regulators [164]. Employing ATAC-seq to analyze chromatin profiles of more than 200,000 single cells in tumors could describe the regulatory program that existed in TEx cells as two stages. Primarily, intermediate TEx was concomitant with the accessibility of NR3C1 and NR4A1 motifs, the direct downstream factors of tumor cell receptor signaling. Secondly, further progression towards terminal TEx was associated with the accessibility of cis-elements near CD101 and TOX genes [165]. Proteomic analysis identified high and sustained TOX expression of TEx in chronic infection and lung cancer [166]. TOX converts continuous antigen stimulation into TEX-specific transcriptional and epigenetic programs, thus becoming Tex commitment initiator and inducing typical exhaustion characteristics [167]. High expression of NR4A transcription factors and enrichment of NR4A binding motifs in chromatin regions was also demonstrated in TEx [168]. Nuclear factor of activated T-cells (NFAT) initiation induced secondary transcription factors of TOX and NR4A family, leading to inhibitory receptors expression to promote TEx. Meanwhile, a positive feedback regulation between NR4A and TOX reinforced this process [169]. Phosphatase PTPN2 was found to be a crucial regulator of differentiation towards terminally exhausted subpopulation based on T cells ATAC-seq and RNA-seq library [170]. TCF-1 also emerged as a developmental catalyst for mature Tex mediating the transcription factors conversion from T-bet to Eomes and driving early fate bifurcation in TEx precursor cells [171, 172].

Multi-omics could be applied to explore TEx molecular mechanisms. Corresponding genetic engineering strategies could reactivate Tex and potentiate CAR-T cell responses. CAR-T cells with a triple knockout of NR4A family members promoted tumor regression and prolonged survival [168]. Double-deficient CAR-tumor-infiltrating lymphocytes (TILs) of TOX and TOX2 showed increased cytokine expression and decreased inhibitory receptor expression, and more efficiently prevented tumor growth than wild-type CAR-T cells [169]. PTPN2‐deficient HER‐2‐targeting CAR-T cells exhibited augmented LCK‐dependent activation and notable suppression of tumor formation. Moreover, it eradicated HER-2 + mammary tumors in vivo, exhibiting remarkable clinical potential [173]. Transcription factors NFAT and its partner AP-1 (Fos-Jun) cooperated to promote T cell function, and in the absence of AP-1, NFAT initiated a negative feedback program that contributed to T cell hyporesponsiveness or exhaustion [140]. Engineering CAR-T cells to overexpress c-Jun (a typical AP-1 factor) increased CAR-T expansion, enhanced effector function, and reduced differentiation towards terminal exhaustion, resulting in a more potent anti-tumor effect [174]. Up-regulated BATF in CAR-T cells was reported to reduce the TOX and inhibitory receptor expression, thereby shielding their phenotype and transcriptional profile from exhaustion, and potentiating effector functions [140].

Immune checkpoints are essential receptors and ligands on co-stimulatory and inhibitory pathways that modulate immune responses [175]. In physiological conditions, immune checkpoints help maintain self-tolerance and regulate the intensity and duration of immune response to avoid autoimmune and tissue damage. However, tumors develop immune resistance through certain immune checkpoint pathways. Multiple immune checkpoints play key roles in TEx. Currently, combining CAR-T therapy with ICB could be effective against TEx. Notably, exhausted T cells under chronic stimulation exhibited enhanced and sustained expression of PD-1 [176]. The TEx feature favors the development of ICB therapy targeting PD-1/PD-L1 to restore the effector functions of CD8 + T cells in tumor models. Blocking PD-1/PDL-1 pathway by PD-1 antibody checkpoint blockade, cell-intrinsic PD-1 shRNA blockade, or a PD-1 dominant negative receptor could reactivate CD28 + CAR-T cells effector function [177]. In multiple tumor models, including leukaemia, melanoma, and ovarian cancer, CAR-T cells engineered to secrete PD-1-blocking single-chain variable fragments have successfully increased survival [178]. Multi-omics analyses present a potent methodology for elucidating TEx, paving way for CAR-T therapy to overcome obstacles.

## Machine learning (ML) contributing to decoding TIME and *CAR*-T cell

### ML and TIME

Providing information related to spatial-level cellular distribution, co-organization, and cell–cell interaction in the TIME, machine learning (ML) methods could advance our understanding of spatio-temporal heterogeneity and complex molecular structures of TIME [20, 179, 180]. ML and artificial intelligence-driven analyses of pathology images enables histological image-level spatial analysis and spatial TIME analysis at the single cell level. In histological image-level, ML analysis allows precise identification and quantification of TILs and their co-organization with neighboring cancer cells. Diagnostic whole-slide image (WSI) analysis is widely used to analyze TIME. Xu et al*.* [181] developed computational approaches using WSI to predict bladder cancer patients' tumor mutation burden (TMB) status and TILs distribution, and then identify spatial heterogeneity and organization in regions with high TMB and TILs, as well as the prognostic effect. Irrespective of cell-scale detail, the ML-driven histological image-level analyses can efficiently provide spatial immune features that will help predict patient prognosis, cancer subtype, treatment response, recurrence, and metastasis. As mentioned above, the distance and distribution of tumor/immune cells are major characteristics of TIME. At the single-cell level, Image analysis algorithms and deep learning (DL) models can be employed to identify and label different nucleus types, such as tumor cell, lymphocyte, and stromal cell nuclei. Via graphic tessellation or hot spot analysis, the nuclear densities, spatial organization, and interactions can be quantified. Some researchers conduct automated histology images and spatial statistical analyses in ovarian cancer pathology slides [179]. Among the Immunoreactive subtype, the spatial analysis shows significantly lower lymphocytic infiltration within diversified zones compared with other tumor zones, suggesting that even I-I TIMEs contain cells capable of immune escape.

### ML and *CAR*-T cell

#### *CAR* construction

CAR constructions steer the phenotypic output and cell fates of therapeutic T cells. The co-stimulatory domains in CARs are composed of multiple signaling motifs, short peptides that bind to specific downstream signaling proteins. Therefore, such peptide signaling motifs can be referred to as the fundamental unit controlling the output of most phenotype of CAR-T cells. Currently, the major goal is still to predictably generate desirable T cell phenotypes by altering receptor construction. Although several specific natural immune receptors have been screened to enhance T cell anti-tumor cytotoxicity or prolong the T cell lifespan, this approach is not effective enough and limited to the currently discovered receptor structures [182, 183]. An effective solution is to screen the natural receptor composition, create a signal motif library, and systematically encode new receptors with different motif identities, combinations and orders. Random combinations of signaling motifs could, in principle, yield phenotypes beyond those that can be generated by native receptor domains alone. Accordingly, those manually encoded CARs can confer novel phenotypes on T cells. Due to the huge size and complexity of the combinatorial space, ML is proper to decode the combinatorial grammar of CAR and guide the design of non-natural co-stimulatory domains with improved phenotypes. Based on 13 signal motifs, Daniels and colleagues constructed a library of CARs containing 2,300 synthetic costimulatory domains [184]. With an arrayed screen of several hundred receptors and machine learning, they identified a non-native combination of motifs that bind tumor necrosis factor receptor-associated factors (TRAFs) and phospholipase C gamma 1 (PLCγ1). Subsequent neural networks predict this combination enhances cytotoxicity and stemness associated with effective tumor killing.

#### Neoantigen

Immunogenic neoantigens have been reported as crucial targets for adoptive T-cell therapies. However, the process of neoantigen discovery and validation remains a formidable question. The emergence of state-of-the-art ML algorithms enabled the identification of T-cell neoantigens through MHC class I/II presentations. The newly developed pipeline utilizes genomics data of tumor samples, usually derived from whole-genome sequencing (WGS) or WES, to infer the mutated peptides based on the somatic non-synonymous single-nucleotide variants (SNVs) [180]. Some other studies employ ML models to predict neoantigens by estimating the binding affinity between a given mutated peptide and an MHC class I molecule [185, 186]. CD4 + T cells recognize antigens presented by class II MHC molecules. Compared to class I MHC molecules, class II MHC molecules are highly polymorphic and the size of the peptides presented is promiscuous, making neoantigen prediction more challenging [187]. Thus, the MHC class II prediction models were generally trained on more complicated datasets [188, 189].

Assessment of binding affinity between the mutant peptide and MHC alone is not sufficient to reliably predict neoantigens, immunogenicity of peptide-MHC (pMHC) also needs to be taken into account. Immunogenicity refers to the ability of a protein product to trigger an immune response. It is related to factors such as protein expression, pMHC binding affinity and stability [190]. DeepHLApan is a deep learning approach for neoantigen prediction considering both the possibility of MHC-peptide binding and the potential immunogenicity of pMHC [191].

### ML and prediction of immunotherapy response

The CAR-T treatment inevitably imposes significant financial, physical, and psychological burdens on patients, so it is critical to predict immunotherapy outcomes and identify patients with therapeutic benefits. Tumors are caused by the accumulation of various genetic variants that regulate how cells grow and proliferate [192]. From this perspective, it is reasonable to predict a patient's response to immunotherapy by exploiting tumor-related genomic biomarkers and molecular characteristics, such as somatic mutations (single-nucleotide variants, insertions, and deletions) [193], pathway activity [194], cell–cell communication [195]. Given the complex omics space, conducting extensive research through experimental methods is impractical. In silico approaches, especially ML algorithms, provide opportunities to address this critical requirement [196, 197].

With the advancement of omics technology, recent research has focused on developing ML prediction models incorporating multi-omics datasets for immunotherapy prediction. The integration of multi-omics data can provide a more comprehensive understanding of tumor conditions, from the original cause to pathological outcomes, thereby improving predictive performance [197, 198].

## Perspective and conclusion

Despite the significant advancements in immunotherapy, some patients persistently encounter suboptimal clinical outcomes. The complexity and heterogeneity of TIME impose considerable constraints on the clinical efficacy of immunotherapy. Multi-omics analyses, including genomics, epigenomics, transcriptomics, proteomics, and microbiomics, have been employed to conduct in-depth investigations on TIME and renovate valuable novel insights into TIME. CAR-T cell therapy is the most anticipated immunotherapies. Nevertheless, application and toxicity remain a Gordian knot. Here, precise and feasible strategies were provided to address these unfavorable conditions from an innovative perspective of multi-dimensional technology.

Commencing with transcriptome, single-cell technology has extended to multi-omics strategies and enabled comprehensive multi-dimensional analysis of bulk sequencing data, providing rich and high-resolution information on all facets of the immune milieu and response in TIME. The future of tumor therapy will inevitably move towards individualization. To create an optimal management strategy tailored to individual cancer patients, multi-dimensional characteristics are indispensable [199]. The combination of single-cell technologies with multi-omics seems to hold promise for tumor therapy, but integrating extensive and intricate multi-dimensional data into biological models and mechanisms is still a considerable challenge. ML is currently the preferred method to address this task. Nevertheless, there is a need to further understand the principles of data integration and visualization methods, and optimize the computational framework for omics data integration so that multi-omics data could be fully utilized to decode TIME and CAR-T immunotherapies.

## Data Availability

Not applicable.

## Abbreviations

TIME: Tumor immune microenvironment
TME: Tumor microenvironment
TLS: Tertiary lymphoid structure
scRNA-seq: Single-cell RNA sequencing
CAR: Chimeric antigen receptor
CTL: Cytotoxic lymphocyte
I–E: Infiltrated–excluded
I-I: Infiltrated-inflamed
ICI: Immune checkpoint inhibitor
HID: Hypothesised Interaction Distribution
CRC: Colorectal cancer
DSS: Disease-specific survival
TGF-β: Transforming growth factor-β
IL1B: Interleukin 1B
MS: Mass spectrometry
EV: Extracellular vesicle
TAM: Tumor-associated macrophage
MDSC: Myeloid-derived suppressor cell
GPC2: Glypican 2
EGFRvIII: Epidermal growth factor receptor variant III
WES: Whole-exome sequencing
NGS: Next-generation sequencing
ECM: Extracellular matrix
EC: Endothelial cell
GC–MS: Gas chromatograph-mass spectrometer
LC–MS: Liquid Chromatograph-Mass Spectrometer
NMR: Nuclear Magnetic Resonance
DON: 6-Diazo-5-oxo-l-norleucine
ADA: Adenosine deaminase 1
COX: Cyclooxygenase
cDC: Conventional dendritic cell
TEx: T cell exhaustion
NFAT: Nuclear factor of activated T-cell
CTLA-4: Cytotoxic T lymphocyte-associated antigen 4
PD-1: Programmed death-1
LAG-3: Lymphocyte activation gene-3
Tim-3: T cell immunoglobulin and mucin-domain containing-3
PD-L1: PD-1 ligand
EGFR: Epidermal growth factor receptor
TNF-α: Tumor necrosis factor-α
CyTOF: Cytometry by Time-Of-Flight
Treg: Regulatory T cell
TIL: Tumor-infiltrating lymphocytes
ML: Machine learning
WSI: Whole-slide image
TMB: Tumor mutation burden
DL: Deep learning
TRAFs: Tumor necrosis factor receptor-associated factors
PLCγ1: Phospholipase C gamma 1
WGS: Whole-genome sequencing
SNV: Single-nucleotide variants
pMHC: Peptide-MHC

## References

[1] Ladanyi A, Timar J (2020). Immunologic and immunogenomic aspects of tumor progression. Semin Cancer Biol.

[2] Binnewies M, Roberts EW, Kersten K, Chan V, Fearon DF, Merad M, Coussens LM, Gabrilovich DI, Ostrand-Rosenberg S, Hedrick CC, Vonderheide RH, Pittet MJ, Jain RK (2018). Understanding the tumor immune microenvironment (TIME) for effective therapy. Nat Med.

[3] Huang W, Jiang Y, Xiong W, Sun Z, Chen C, Yuan Q, Zhou K, Han Z, Feng H, Chen H, Liang X, Yu S, Hu Y (2022). Noninvasive imaging of the tumor immune microenvironment correlates with response to immunotherapy in gastric cancer. Nat Commun.

[4] Baharom F, Ramirez-Valdez RA, Khalilnezhad A, Khalilnezhad S, Dillon M, Hermans D, Fussell S, Tobin KKS, Dutertre CA, Lynn GM, Muller S, Ginhoux F, Ishizuka AS (2022). Systemic vaccination induces CD8(+) T cells and remodels the tumor microenvironment. Cell.

[5] Liu Z, Zhou Z, Dang Q, Xu H, Lv J, Li H, Han X (2022). Immunosuppression in tumor immune microenvironment and its optimization from CAR-T cell therapy. Theranostics.

[6] Schumacher TN, Thommen DS (2022). Tertiary lymphoid structures in cancer. Science.

[7] Newman AM, Liu CL, Green MR, Gentles AJ, Feng W, Xu Y, Hoang CD, Diehn M, Alizadeh AA (2015). Robust enumeration of cell subsets from tissue expression profiles. Nat Methods.

[8] Aran D, Hu Z, Butte AJ (2017). xCell: digitally portraying the tissue cellular heterogeneity landscape. Genome Biol.

[9] Olivier M, Asmis R, Hawkins GA, Howard TD, Cox LA. The Need for Multi-Omics Biomarker Signatures in Precision Medicine. Int J Mol Sci. 2019;20(19):4781. 10.3390/ijms20194781 PMC6801754 31561483

[10] Raufaste-Cazavieille V, Santiago R, Droit A (2022). Multi-omics analysis: Paving the path toward achieving precision medicine in cancer treatment and immuno-oncology. Front Mol Biosci.

[11] Mavi AK, Gaur S, Gaur G, Babita, Kumar N, Kumar U (2023). CAR T-cell therapy: Reprogramming patient's immune cell to treat cancer. Cell Signal.

[12] Roselli E, Boucher JC, Li G, Kotani H, Spitler K, Reid K, Cervantes EV, Bulliard Y, Tu N, Lee SB, Yu B, Locke FL, Davila ML. 4–1BB and optimized CD28 co-stimulation enhances function of human mono-specific and bi-specific third-generation CAR T cells. J Immunother Cancer. 2021;9(10):e003354. 10.1136/jitc-2021-003354 PMC8552146 34706886

[13] Li W, Zhou Y, Wu Z, Shi Y, Tian E, Zhu Y, Wang T, Dou W, Meng X, Chen M, Zhai B, Zhu D (2021). Targeting Wnt Signaling in the Tumor Immune Microenvironment to Enhancing EpCAM CAR T-Cell therapy. Front Pharmacol.

[14] Yang J, Chen Y, Han L (2023). A multi-omics perspective of CAR T cell therapy. Clin Transl Med.

[15] Spranger S (2016). Mechanisms of tumor escape in the context of the T-cell-inflamed and the non-T-cell-inflamed tumor microenvironment. Int Immunol.

[16] Evans RA, Diamond MS, Rech AJ, Chao T, Richardson MW, Lin JH, Bajor DL, Byrne KT, Stanger BZ, Riley JL, Markosyan N, Winograd R, Vonderheide RH. Lack of immunoediting in murine pancreatic cancer reversed with neoantigen. JCI Insight. 2016;1(14):e88328. 10.1172/jci.insight.88328 PMC5026128 27642636

[17] Lee HJ, Park IA, Song IH, Shin SJ, Kim JY, Yu JH, Gong G. Tertiary lymphoid structures: prognostic significance and relationship with tumour-infiltrating lymphocytes in triple-negative breast cancer. J Clin Pathol. 2016;69:422–30. 10.1136/jclinpath-2015-203089 26475777

[18] Sautes-Fridman C, Lawand M, Giraldo NA, Kaplon H, Germain C, Fridman WH, Dieu-Nosjean MC (2016). Tertiary Lymphoid Structures in Cancers: Prognostic Value, Regulation, and Manipulation for Therapeutic Intervention. Front Immunol.

[19] Finkin S, Yuan D, Stein I, Taniguchi K, Weber A, Unger K, Browning JL, Goossens N, Nakagawa S, Gunasekaran G, Schwartz ME, Kobayashi M, Kumada H (2015). Ectopic lymphoid structures function as microniches for tumor progenitor cells in hepatocellular carcinoma. Nat Immunol.

[20] Xu H, Cong F, Hwang TH (2021). Machine Learning and Artificial Intelligence-driven Spatial Analysis of the Tumor Immune Microenvironment in Pathology Slides. Eur Urol Focus.

[21] Tsujikawa T, Mitsuda J, Ogi H, Miyagawa-Hayashino A, Konishi E, Itoh K, Hirano S (2020). Prognostic significance of spatial immune profiles in human solid cancers. Cancer Sci.

[22] Gruosso T, Gigoux M, Manem VSK, Bertos N, Zuo D, Perlitch I, Saleh SMI, Zhao H, Souleimanova M, Johnson RM, Monette A, Ramos VM, Hallett MT (2019). Spatially distinct tumor immune microenvironments stratify triple-negative breast cancers. J Clin Invest.

[23] Phanthunane C, Wijers R, de Herdt M, Langeveld TPM, Koljenovic S, Dasgupta S, Sleijfer S, de Baatenburg Jong RJ, Hardillo J, Balcioglu HE, Debets R (2021). B-cell clusters at the invasive margin associate with longer survival in early-stage oral-tongue cancer patients. Oncoimmunology.

[24] Glajcar A, Szpor J, Pacek A, Tyrak KE, Chan F, Streb J, Hodorowicz-Zaniewska D, Okon K (2017). The relationship between breast cancer molecular subtypes and mast cell populations in tumor microenvironment. Virchows Arch.

[25] Berthel A, Zoernig I, Valous NA, Kahlert C, Klupp F, Ulrich A, Weitz J, Jaeger D, Halama N (2017). Detailed resolution analysis reveals spatial T cell heterogeneity in the invasive margin of colorectal cancer liver metastases associated with improved survival. Oncoimmunology.

[26] Wu Y, Cheng Y, Wang X, Fan J, Gao Q (2022). Spatial omics: Navigating to the golden era of cancer research. Clin Transl Med.

[27] Halle S, Halle O, Forster R (2017). Mechanisms and Dynamics of T Cell-Mediated Cytotoxicity In Vivo. Trends Immunol.

[28] Liu M, Kuo F, Capistrano KJ, Kang D, Nixon BG, Shi W, Chou C, Do MH, Stamatiades EG, Gao S, Li S, Chen Y, Hsieh JJ (2020). TGF-beta suppresses type 2 immunity to cancer. Nature.

[29] Kim HD, Kim JH, Ryu YM, Kim D, Lee S, Shin J, Hong SM, Kim KH, Jung DH, Song GW, Hwang DW, Lee JH, Song KB (2021). Spatial Distribution and Prognostic Implications of Tumor-Infiltrating FoxP3- CD4+ T Cells in Biliary Tract Cancer. Cancer Res Treat.

[30] Datar I, Sanmamed MF, Wang J, Henick BS, Choi J, Badri T, Dong W, Mani N, Toki M, Mejias LD, Lozano MD, Perez-Gracia JL, Velcheti V (2019). Expression Analysis and Significance of PD-1, LAG-3, and TIM-3 in Human Non-Small Cell Lung Cancer Using Spatially Resolved and Multiparametric Single-Cell Analysis. Clin Cancer Res.

[31] Tsakiroglou AM, Fergie M, Oguejiofor K, Linton K, Thomson D, Stern PL, Astley S, Byers R, West CML (2020). Spatial proximity between T and PD-L1 expressing cells as a prognostic biomarker for oropharyngeal squamous cell carcinoma. Br J Cancer.

[32] Giraldo NA, Nguyen P, Engle EL, Kaunitz GJ, Cottrell TR, Berry S, Green B, Soni A, Cuda JD, Stein JE, Sunshine JC, Succaria F, Xu H (2018). Multidimensional, quantitative assessment of PD-1/PD-L1 expression in patients with Merkel cell carcinoma and association with response to pembrolizumab. J Immunother Cancer.

[33] Johnson DB, Bordeaux J, Kim JY, Vaupel C, Rimm DL, Ho TH, Joseph RW, Daud AI, Conry RM, Gaughan EM, Hernandez-Aya LF, Dimou A, Funchain P (2018). Quantitative Spatial Profiling of PD-1/PD-L1 Interaction and HLA-DR/IDO-1 Predicts Improved Outcomes of Anti-PD-1 Therapies in Metastatic Melanoma. Clin Cancer Res.

[34] Griffith BD, Turcotte S, Lazarus J, Lima F, Bell S, Delrosario L, McGue J, Krishnan S, Oneka MD, Nathan H, Smith JJ, D'Angelica MI, Shia J, et al. MHC Class II Expression Influences the Composition and Distribution of Immune Cells in the Metastatic Colorectal Cancer Microenvironment. Cancers (Basel). 2022;14(17):4092. 10.3390/cancers14174092 PMC9454847 36077630

[35] Fu T, Dai LJ, Wu SY, Xiao Y, Ma D, Jiang YZ, Shao ZM (2021). Spatial architecture of the immune microenvironment orchestrates tumor immunity and therapeutic response. J Hematol Oncol.

[36] Parra ER, Ferrufino-Schmidt MC, Tamegnon A, Zhang J, Solis L, Jiang M, Ibarguen H, Haymaker C, Lee JJ, Bernatchez C, Wistuba II (2021). Immuno-profiling and cellular spatial analysis using five immune oncology multiplex immunofluorescence panels for paraffin tumor tissue. Sci Rep.

[37] Tien TZ, Lee J, Lim JCT, Chen XY, Thike AA, Tan PH, Yeong JPS (2021). Delineating the breast cancer immune microenvironment in the era of multiplex immunohistochemistry/immunofluorescence. Histopathology.

[38] Schwen LO, Andersson E, Korski K, Weiss N, Haase S, Gaire F, Hahn HK, Homeyer A, Grimm O (2018). Data-Driven Discovery of Immune Contexture Biomarkers. Front Oncol.

[39] Nearchou IP, Lillard K, Gavriel CG, Ueno H, Harrison DJ, Caie PD (2019). Automated Analysis of Lymphocytic Infiltration, Tumor Budding, and Their Spatial Relationship Improves Prognostic Accuracy in Colorectal Cancer. Cancer Immunol Res.

[40] Gartrell RD, Marks DK, Hart TD, Li G, Davari DR, Wu A, Blake Z, Lu Y, Askin KN, Monod A, Esancy CL, Stack EC, Jia DT (2018). Quantitative Analysis of Immune Infiltrates in Primary Melanoma. Cancer Immunol Res.

[41] Gide TN, Silva IP, Quek C, Ahmed T, Menzies AM, Carlino MS, Saw RPM, Thompson JF, Batten M, Long GV, Scolyer RA, Wilmott JS (2020). Close proximity of immune and tumor cells underlies response to anti-PD-1 based therapies in metastatic melanoma patients. Oncoimmunology.

[42] Lundgren S, Elebro J, Heby M, Nodin B, Leandersson K, Micke P, Jirstrom K, Mezheyeuski A (2020). Quantitative, qualitative and spatial analysis of lymphocyte infiltration in periampullary and pancreatic adenocarcinoma. Int J Cancer.

[43] Feichtenbeiner A, Haas M, Buttner M, Grabenbauer GG, Fietkau R, Distel LV (2014). Critical role of spatial interaction between CD8(+) and Foxp3(+) cells in human gastric cancer: the distance matters. Cancer Immunol Immunother.

[44] Nagl S, Haas M, Lahmer G, Buttner-Herold M, Grabenbauer GG, Fietkau R, Distel LV (2016). Cell-to-cell distances between tumor-infiltrating inflammatory cells have the potential to distinguish functionally active from suppressed inflammatory cells. Oncoimmunology.

[45] Chen DS, Mellman I (2017). Elements of cancer immunity and the cancer-immune set point. Nature.

[46] de Visser KE, Joyce JA (2023). The evolving tumor microenvironment: From cancer initiation to metastatic outgrowth. Cancer Cell.

[47] Gajewski TF, Schreiber H, Fu YX (2013). Innate and adaptive immune cells in the tumor microenvironment. Nat Immunol.

[48] Claussnitzer M, Cho JH, Collins R, Cox NJ, Dermitzakis ET, Hurles ME, Kathiresan S, Kenny EE, Lindgren CM, MacArthur DG, North KN, Plon SE, Rehm HL (2020). A brief history of human disease genetics. Nature.

[49] International Multiple Sclerosis Genetics C (2019). A systems biology approach uncovers cell-specific gene regulatory effects of genetic associations in multiple sclerosis. Nat Commun.

[50] International Multiple Sclerosis Genetics C. Multiple sclerosis genomic map implicates peripheral immune cells and microglia in susceptibility. Science. 2019;365(6460):eaav7188. 10.1126/science.aav7188 PMC7241648 31604244

[51] Vuckovic D, Bao EL, Akbari P, Lareau CA, Mousas A, Jiang T, Chen MH, Raffield LM, Tardaguila M, Huffman JE, Ritchie SC, Megy K, Ponstingl H (2020). The Polygenic and Monogenic Basis of Blood Traits and Diseases. Cell.

[52] Orru V, Steri M, Sidore C, Marongiu M, Serra V, Olla S, Sole G, Lai S, Dei M, Mulas A, Virdis F, Piras MG, Lobina M (2020). Complex genetic signatures in immune cells underlie autoimmunity and inform therapy. Nat Genet.

[53] Patin E, Hasan M, Bergstedt J, Rouilly V, Libri V, Urrutia A, Alanio C, Scepanovic P, Hammer C, Jonsson F, Beitz B, Quach H, Lim YW (2018). Natural variation in the parameters of innate immune cells is preferentially driven by genetic factors. Nat Immunol.

[54] Sayaman RW, Saad M, Thorsson V, Hu D, Hendrickx W, Roelands J, Porta-Pardo E, Mokrab Y, Farshidfar F, Kirchhoff T, Sweis RF, Bathe OF, Heimann C (2021). Germline genetic contribution to the immune landscape of cancer. Immunity.

[55] Lim YW, Chen-Harris H, Mayba O, Lianoglou S, Wuster A, Bhangale T, Khan Z, Mariathasan S, Daemen A, Reeder J, Haverty PM, Forrest WF, Brauer M (2018). Germline genetic polymorphisms influence tumor gene expression and immune cell infiltration. Proc Natl Acad Sci U S A.

[56] Cursons J, Souza-Fonseca-Guimaraes F, Foroutan M, Anderson A, Hollande F, Hediyeh-Zadeh S, Behren A, Huntington ND, Davis MJ (2019). A Gene Signature Predicting Natural Killer Cell Infiltration and Improved Survival in Melanoma Patients. Cancer Immunol Res.

[57] Nabbi A, Danesh A, Espin-Garcia O, Pedersen S, Wellum J, Fu LH, Paulson JN, Geoerger B, Marshall LV, Trippett T, Rossato G, Pugh TJ, Hutchinson KE (2023). Multimodal immunogenomic biomarker analysis of tumors from pediatric patients enrolled to a phase 1–2 study of single-agent atezolizumab. Nat Cancer.

[58] Addeo A, Passaro A, Malapelle U, Banna GL, Subbiah V, Friedlaender A (2021). Immunotherapy in non-small cell lung cancer harbouring driver mutations. Cancer Treat Rev.

[59] Marabelle A, Le DT, Ascierto PA, Di Giacomo AM, De Jesus-Acosta A, Delord JP, Geva R, Gottfried M, Penel N, Hansen AR, Piha-Paul SA, Doi T, Gao B (2020). Efficacy of Pembrolizumab in Patients With Noncolorectal High Microsatellite Instability/Mismatch Repair-Deficient Cancer: Results From the Phase II KEYNOTE-158 Study. J Clin Oncol..

[60] Lam H, McNeil LK, Starobinets H, DeVault VL, Cohen RB, Twardowski P, Johnson ML, Gillison ML, Stein MN, Vaishampayan UN, DeCillis AP, Foti JJ, Vemulapalli V (2021). An Empirical Antigen Selection Method Identifies Neoantigens That Either Elicit Broad Antitumor T-cell Responses or Drive Tumor Growth. Cancer Discov.

[61] Launonen IM, Lyytikainen N, Casado J, Anttila EA, Szabo A, Haltia UM, Jacobson CA, Lin JR, Maliga Z, Howitt BE, Strickland KC, Santagata S, Elias K (2022). Single-cell tumor-immune microenvironment of BRCA1/2 mutated high-grade serous ovarian cancer. Nat Commun.

[62] McGranahan N, Furness AJ, Rosenthal R, Ramskov S, Lyngaa R, Saini SK, Jamal-Hanjani M, Wilson GA, Birkbak NJ, Hiley CT, Watkins TB, Shafi S, Murugaesu N (2016). Clonal neoantigens elicit T cell immunoreactivity and sensitivity to immune checkpoint blockade. Science.

[63] Blass E, Ott PA (2021). Advances in the development of personalized neoantigen-based therapeutic cancer vaccines. Nat Rev Clin Oncol.

[64] Rooney MS, Shukla SA, Wu CJ, Getz G, Hacohen N (2015). Molecular and genetic properties of tumors associated with local immune cytolytic activity. Cell.

[65] Sun L, Zhang H, Gao P (2022). Metabolic reprogramming and epigenetic modifications on the path to cancer. Protein Cell.

[66] Quezada LK, Jin W, Liu YC, Kim ES, He Z, Indralingam CS, Tysl T, Labarta-Bajo L, Wehrens EJ, Jo Y, Kazane KR, Hattori C, Zuniga EI (2023). Early transcriptional and epigenetic divergence of CD8+ T cells responding to acute versus chronic infection. PLoS Biol.

[67] Li R, Ong SL, Tran LM, Jing Z, Liu B, Park SJ, Huang ZL, Walser TC, Heinrich EL, Lee G, Salehi-Rad R, Crosson WP, Pagano PC (2020). Chronic IL-1beta-induced inflammation regulates epithelial-to-mesenchymal transition memory phenotypes via epigenetic modifications in non-small cell lung cancer. Sci Rep.

[68] Tekpli X, Landvik NE, Anmarkud KH, Skaug V, Haugen A, Zienolddiny S (2013). DNA methylation at promoter regions of interleukin 1B, interleukin 6, and interleukin 8 in non-small cell lung cancer. Cancer Immunol Immunother.

[69] Asgarova A, Asgarov K, Godet Y, Peixoto P, Nadaradjane A, Boyer-Guittaut M, Galaine J, Guenat D, Mougey V, Perrard J, Pallandre JR, Bouard A, Balland J (2018). PD-L1 expression is regulated by both DNA methylation and NF-kB during EMT signaling in non-small cell lung carcinoma. Oncoimmunology.

[70] Chaves LP, Melo CM, Lautert-Dutra W, Caliari AL, Squire JA, Passos GA (2022). Trannscriptomics and Immune Response in Human Cancer. Transcriptomics in Health and Disease.

[71] Zhang Q, Gao C, Shao J, Wang Z. TIGIT-related transcriptome profile and its association with tumor immune microenvironment in breast cancer. Biosci Rep. 2021;41(3):BSR20204340. 10.1042/BSR20204340 PMC7990089 33721026

[72] Skafi N, Fayyad-Kazan M, Badran B (2020). Immunomodulatory role for MicroRNAs: Regulation of PD-1/PD-L1 and CTLA-4 immune checkpoints expression. Gene.

[73] Ren X, Zhang L, Zhang Y, Li Z, Siemers N, Zhang Z (2021). Insights Gained from Single-Cell Analysis of Immune Cells in the Tumor Microenvironment. Annu Rev Immunol.

[74] Tirosh I, Izar B, Prakadan SM, Wadsworth MH, Treacy D, Trombetta JJ, Rotem A, Rodman C, Lian C, Murphy G, Fallahi-Sichani M, Dutton-Regester K, Lin JR (2016). Dissecting the multicellular ecosystem of metastatic melanoma by single-cell RNA-seq. Science.

[75] Pan Y, Lu F, Fei Q, Yu X, Xiong P, Yu X, Dang Y, Hou Z, Lin W, Lin X, Zhang Z, Pan M, Huang H (2019). Single-cell RNA sequencing reveals compartmental remodeling of tumor-infiltrating immune cells induced by anti-CD47 targeting in pancreatic cancer. J Hematol Oncol.

[76] Chen J, Liu K, Luo Y, Kang M, Wang J, Chen G, Qi J, Wu W, Wang B, Han Y, Shi L, Wang K, Han X, et al. Single-Cell Profiling of Tumor Immune Microenvironment Reveals Immune Irresponsiveness in Gastric Signet-Ring Cell Carcinoma. Gastroenterology. 2023;165(1):88–103. 10.1053/j.gastro.2023.03.008 36921674

[77] Zou Y, Ye F, Kong Y, Hu X, Deng X, Xie J, Song C, Ou X, Wu S, Wu L, Xie Y, Tian W, Tang Y (2023). The Single-Cell Landscape of Intratumoral Heterogeneity and The Immunosuppressive Microenvironment in Liver and Brain Metastases of Breast Cancer. Adv Sci (Weinh).

[78] Martinez-Val A, Guzman UH, Olsen JV (2022). Obtaining Complete Human Proteomes. Annu Rev Genomics Hum Genet.

[79] Lehtio J, Arslan T, Siavelis I, Pan Y, Socciarelli F, Berkovska O, Umer HM, Mermelekas G, Pirmoradian M, Jonsson M, Brunnstrom H, Brustugun OT, Purohit KP (2021). Proteogenomics of non-small cell lung cancer reveals molecular subtypes associated with specific therapeutic targets and immune evasion mechanisms. Nat Cancer.

[80] Satpathy S, Krug K, Jean Beltran PM, Savage SR, Petralia F, Kumar-Sinha C, Dou Y, Reva B, Kane MH, Avanessian SC, Vasaikar SV, Krek A, Lei JT (2021). A proteogenomic portrait of lung squamous cell carcinoma. Cell.

[81] Tanzer MC, Bludau I, Stafford CA, Hornung V, Mann M (2021). Phosphoproteome profiling uncovers a key role for CDKs in TNF signaling. Nat Commun.

[82] Bartok O, Pataskar A, Nagel R, Laos M, Goldfarb E, Hayoun D, Levy R, Korner PR, Kreuger IZM, Champagne J, Zaal EA, Bleijerveld OB, Huang X (2021). Anti-tumour immunity induces aberrant peptide presentation in melanoma. Nature.

[83] Cianciaruso C, Beltraminelli T, Duval F, Nassiri S, Hamelin R, Mozes A, Gallart-Ayala H, Ceada Torres G, Torchia B, Ries CH, Ivanisevic J, De Palma M (2019). Molecular Profiling and Functional Analysis of Macrophage-Derived Tumor Extracellular Vesicles. Cell Rep.

[84] Di J, Liu M, Fan Y, Gao P, Wang Z, Jiang B, Su X (2020). Phenotype molding of T cells in colorectal cancer by single-cell analysis. Int J Cancer.

[85] Fu W, Wang W, Li H, Jiao Y, Huo R, Yan Z, Wang J, Wang S, Wang J, Chen D, Cao Y, Zhao J (2020). Single-Cell Atlas Reveals Complexity of the Immunosuppressive Microenvironment of Initial and Recurrent Glioblastoma. Front Immunol.

[86] Sepich-Poore GD, Zitvogel L, Straussman R, Hasty J, Wargo JA, Knight R. The microbiome and human cancer. Science. 2021;371(6536):eabc4552. 10.1126/science.abc4552 PMC8767999 33766858

[87] Zhou CB, Zhou YL, Fang JY (2021). Gut Microbiota in Cancer Immune Response and Immunotherapy. Trends Cancer.

[88] Zitvogel L, Ayyoub M, Routy B, Kroemer G (2016). Microbiome and Anticancer Immunosurveillance. Cell.

[89] Chen Y, Liu B, Wei Y, Kuang DM (2021). Influence of gut and intratumoral microbiota on the immune microenvironment and anti-cancer therapy. Pharmacol Res.

[90] Abreu MT, Peek RM (2014). Gastrointestinal malignancy and the microbiome. Gastroenterology.

[91] Riquelme E, Zhang Y, Zhang L, Montiel M, Zoltan M, Dong W, Quesada P, Sahin I, Chandra V, San Lucas A, Scheet P, Xu H, Hanash SM (2019). Tumor Microbiome Diversity and Composition Influence Pancreatic Cancer Outcomes. Cell.

[92] Yu LX, Schwabe RF (2017). The gut microbiome and liver cancer: mechanisms and clinical translation. Nat Rev Gastroenterol Hepatol.

[93] Overacre-Delgoffe AE, Bumgarner HJ, Cillo AR, Burr AHP, Tometich JT, Bhattacharjee A, Bruno TC, Vignali DAA, Hand TW (2021). Microbiota-specific T follicular helper cells drive tertiary lymphoid structures and anti-tumor immunity against colorectal cancer. Immunity.

[94] Guo C, Guo D, Fang L, Sang T, Wu J, Guo C, Wang Y, Wang Y, Chen C, Chen J, Chen R, Wang X (2021). Ganoderma lucidum polysaccharide modulates gut microbiota and immune cell function to inhibit inflammation and tumorigenesis in colon. Carbohydr Polym.

[95] Pushalkar S, Hundeyin M, Daley D, Zambirinis CP, Kurz E, Mishra A, Mohan N, Aykut B, Usyk M, Torres LE, Werba G, Zhang K, Guo Y (2018). The Pancreatic Cancer Microbiome Promotes Oncogenesis by Induction of Innate and Adaptive Immune Suppression. Cancer Discov.

[96] Brand A, Singer K, Koehl GE, Kolitzus M, Schoenhammer G, Thiel A, Matos C, Bruss C, Klobuch S, Peter K, Kastenberger M, Bogdan C, Schleicher U (2016). LDHA-Associated Lactic Acid Production Blunts Tumor Immunosurveillance by T and NK Cells. Cell Metab.

[97] Greathouse KL, White JR, Vargas AJ, Bliskovsky VV, Beck JA, von Muhlinen N, Polley EC, Bowman ED, Khan MA, Robles AI, Cooks T, Ryan BM, Padgett N (2018). Interaction between the microbiome and TP53 in human lung cancer. Genome Biol.

[98] Jin C, Lagoudas GK, Zhao C, Bullman S, Bhutkar A, Hu B, Ameh S, Sandel D, Liang XS, Mazzilli S, Whary MT, Meyerson M, Germain R (2019). Commensal Microbiota Promote Lung Cancer Development via gammadelta T Cells. Cell.

[99] Grivennikov SI, Wang K, Mucida D, Stewart CA, Schnabl B, Jauch D, Taniguchi K, Yu GY, Osterreicher CH, Hung KE, Datz C, Feng Y, Fearon ER (2012). Adenoma-linked barrier defects and microbial products drive IL-23/IL-17-mediated tumour growth. Nature.

[100] Correction (2020). The Pancreatic Cancer Microbiome Promotes Oncogenesis by Induction of Innate and Adaptive Immune Suppression. Cancer Discov.

[101] Matson V, Chervin CS, Gajewski TF (2021). Cancer and the Microbiome-Influence of the Commensal Microbiota on Cancer, Immune Responses, and Immunotherapy. Gastroenterology.

[102] Sterner RC, Sterner RM (2021). CAR-T cell therapy: current limitations and potential strategies. Blood Cancer J.

[103] Flugel CL, Majzner RG, Krenciute G, Dotti G, Riddell SR, Wagner DL, Abou-El-Enein M (2023). Overcoming on-target, off-tumour toxicity of CAR T cell therapy for solid tumours. Nat Rev Clin Oncol.

[104] Hou AJ, Chen LC, Chen YY (2021). Navigating CAR-T cells through the solid-tumour microenvironment. Nat Rev Drug Discov.

[105] Wei J, Han X, Bo J, Han W (2019). Target selection for CAR-T therapy. J Hematol Oncol.

[106] Leung KK, Wilson GM, Kirkemo LL, Riley NM, Coon JJ, Wells JA (2020). Broad and thematic remodeling of the surfaceome and glycoproteome on isogenic cells transformed with driving proliferative oncogenes. Proc Natl Acad Sci U S A.

[107] Foster JB, Griffin C, Rokita JL, Stern A, Brimley C, Rathi K, Lane MV, Buongervino SN, Smith T, Madsen PJ, Martinez D, Delaidelli A, Sorensen PH, et al. Development of GPC2-directed chimeric antigen receptors using mRNA for pediatric brain tumors. J Immunother Cancer. 2022;10(9):e004450. 10.1136/jitc-2021-004450 PMC9516314 36167467

[108] MacKay M, Afshinnekoo E, Rub J, Hassan C, Khunte M, Baskaran N, Owens B, Liu L, Roboz GJ, Guzman ML, Melnick AM, Wu S, Mason CE (2020). The therapeutic landscape for cells engineered with chimeric antigen receptors. Nat Biotechnol.

[109] Liu Z, Lv J, Dang Q, Liu L, Weng S, Wang L, Zhou Z, Kong Y, Li H, Han Y, Han X (2022). Engineering neoantigen vaccines to improve cancer personalized immunotherapy. Int J Biol Sci.

[110] Buonaguro L, Tagliamonte M. Selecting Target Antigens for Cancer Vaccine Development. Vaccines (Basel). 2020;8(4):615. 10.3390/vaccines8040615 PMC7711972 33080888

[111] Xie N, Shen G, Gao W, Huang Z, Huang C, Fu L (2023). Neoantigens: promising targets for cancer therapy. Signal Transduct Target Ther.

[112] Wang Z, Cao YJ (2020). Adoptive Cell Therapy Targeting Neoantigens: A Frontier for Cancer Research. Front Immunol.

[113] Zhou C, Zhu C, Liu Q (2019). Toward in silico Identification of Tumor Neoantigens in Immunotherapy. Trends Mol Med.

[114] Gros A, Tran E, Parkhurst MR, Ilyas S, Pasetto A, Groh EM, Robbins PF, Yossef R, Garcia-Garijo A, Fajardo CA, Prickett TD, Jia L, Gartner JJ (2019). Recognition of human gastrointestinal cancer neoantigens by circulating PD-1+ lymphocytes. J Clin Invest.

[115] Shen CJ, Yang YX, Han EQ, Cao N, Wang YF, Wang Y, Zhao YY, Zhao LM, Cui J, Gupta P, Wong AJ, Han SY (2013). Chimeric antigen receptor containing ICOS signaling domain mediates specific and efficient antitumor effect of T cells against EGFRvIII expressing glioma. J Hematol Oncol.

[116] Posey AD, Schwab RD, Boesteanu AC, Steentoft C, Mandel U, Engels B, Stone JD, Madsen TD, Schreiber K, Haines KM, Cogdill AP, Chen TJ, Song D (2016). Engineered CAR T Cells Targeting the Cancer-Associated Tn-Glycoform of the Membrane Mucin MUC1 Control Adenocarcinoma. Immunity.

[117] Jing Y, Liu Y, Li Q, Ye Y, Diao L, Huang Y, Zhou Y, Green MR, Mills GB, Han L (2021). Expression of chimeric antigen receptor therapy targets detected by single-cell sequencing of normal cells may contribute to off-tumor toxicity. Cancer Cell.

[118] Yang J, Chen Y, Jing Y, Green MR, Han L (2023). Advancing CAR T cell therapy through the use of multidimensional omics data. Nat Rev Clin Oncol.

[119] Perna F, Berman SH, Soni RK, Mansilla-Soto J, Eyquem J, Hamieh M, Hendrickson RC, Brennan CW, Sadelain M (2017). Integrating Proteomics and Transcriptomics for Systematic Combinatorial Chimeric Antigen Receptor Therapy of AML. Cancer Cell.

[120] Hu Z, Yuan J, Long M, Jiang J, Zhang Y, Zhang T, Xu M, Fan Y, Tanyi JL, Montone KT, Tavana O, Chan HM, Hu X (2021). The Cancer Surfaceome Atlas integrates genomic, functional and drug response data to identify actionable targets. Nat Cancer.

[121] Dannenfelser R, Allen GM, VanderSluis B, Koegel AK, Levinson S, Stark SR, Yao V, Tadych A, Troyanskaya OG, Lim WA (2020). Discriminatory Power of Combinatorial Antigen Recognition in Cancer T Cell Therapies. Cell Syst.

[122] Beckermann KE, Dudzinski SO, Rathmell JC (2017). Dysfunctional T cell metabolism in the tumor microenvironment. Cytokine Growth Factor Rev.

[123] Razavi AS, Loskog A, Razi S, Rezaei N (2023). The signaling and the metabolic differences of various CAR T cell designs. Int Immunopharmacol.

[124] Wishart DS (2019). Metabolomics for Investigating Physiological and Pathophysiological Processes. Physiol Rev.

[125] Huang Y, Si X, Shao M, Teng X, Xiao G, Huang H (2022). Rewiring mitochondrial metabolism to counteract exhaustion of CAR-T cells. J Hematol Oncol.

[126] Vardhana SA, Hwee MA, Berisa M, Wells DK, Yost KE, King B, Smith M, Herrera PS, Chang HY, Satpathy AT, van den Brink MRM, Cross JR, Thompson CB (2020). Impaired mitochondrial oxidative phosphorylation limits the self-renewal of T cells exposed to persistent antigen. Nat Immunol.

[127] Shen L, Xiao Y, Zhang C, Li S, Teng X, Cui L, Liu T, Wu N, Lu Z (2022). Metabolic reprogramming by ex vivo glutamine inhibition endows CAR-T cells with less-differentiated phenotype and persistent antitumor activity. Cancer Lett.

[128] Kawalekar OU, O'Connor RS, Fraietta JA, Guo L, McGettigan SE, Posey AD, Patel PR, Guedan S, Scholler J, Keith B, Snyder NW, Blair IA, Milone MC (2016). Distinct Signaling of Coreceptors Regulates Specific Metabolism Pathways and Impacts Memory Development in CAR T Cells. Immunity.

[129] Qu Y, Dunn ZS, Chen X, MacMullan M, Cinay G, Wang HY, Liu J, Hu F, Wang P (2022). Adenosine Deaminase 1 Overexpression Enhances the Antitumor Efficacy of Chimeric Antigen Receptor-Engineered T Cells. Hum Gene Ther.

[130] Renauer P, Park JJ, Bai M, Acosta A, Lee WH, Lin GH, Zhang Y, Dai X, Wang G, Errami Y, Wu T, Clark P, Ye L, et al. Immunogenetic metabolomics revealed key enzymes that modulate CAR-T metabolism and function. Cancer Immunol Res. 2023;11(8):1068–84. 10.1158/2326-6066.CIR-22-0565 PMC10527769 37253111

[131] Ye L, Park JJ, Peng L, Yang Q, Chow RD, Dong MB, Lam SZ, Guo J, Tang E, Zhang Y, Wang G, Dai X, Du Y (2022). A genome-scale gain-of-function CRISPR screen in CD8 T cells identifies proline metabolism as a means to enhance CAR-T therapy. Cell Metab.

[132] Quail DF, Joyce JA (2013). Microenvironmental regulation of tumor progression and metastasis. Nat Med.

[133] Costa A, Thirant C, Kramdi A, Pierre-Eugene C, Louis-Brennetot C, Blanchard O, Surdez D, Gruel N, Lapouble E, Pierron G, Sitbon D, Brisse H, Gauthier A, et al. Single-cell transcriptomics reveals shared immunosuppressive landscapes of mouse and human neuroblastoma. J Immunother Cancer. 2022;10(8):e004807. 10.1136/jitc-2022-004807 PMC9362821 36054452

[134] Steele CW, Karim SA, Leach JDG, Bailey P, Upstill-Goddard R, Rishi L, Foth M, Bryson S, McDaid K, Wilson Z, Eberlein C, Candido JB, Clarke M (2016). CXCR2 Inhibition Profoundly Suppresses Metastases and Augments Immunotherapy in Pancreatic Ductal Adenocarcinoma. Cancer Cell.

[135] Greene S, Robbins Y, Mydlarz WK, Huynh AP, Schmitt NC, Friedman J, Horn LA, Palena C, Schlom J, Maeda DY, Zebala JA, Clavijo PE, Allen C (2020). Inhibition of MDSC Trafficking with SX-682, a CXCR1/2 Inhibitor, Enhances NK-Cell Immunotherapy in Head and Neck Cancer Models. Clin Cancer Res.

[136] Sun L, Clavijo PE, Robbins Y, Patel P, Friedman J, Greene S, Das R, Silvin C, Van Waes C, Horn LA, Schlom J, Palena C, Maeda D, et al. Inhibiting myeloid-derived suppressor cell trafficking enhances T cell immunotherapy. JCI Insight. 2019;4(7):e126853. 10.1172/jci.insight.126853 PMC6483637 30944253

[137] Burga RA, Thorn M, Point GR, Guha P, Nguyen CT, Licata LA, DeMatteo RP, Ayala A, Joseph Espat N, Junghans RP, Katz SC (2015). Liver myeloid-derived suppressor cells expand in response to liver metastases in mice and inhibit the anti-tumor efficacy of anti-CEA CAR-T. Cancer Immunol Immunother.

[138] Fujita M, Kohanbash G, Fellows-Mayle W, Hamilton RL, Komohara Y, Decker SA, Ohlfest JR, Okada H (2011). COX-2 blockade suppresses gliomagenesis by inhibiting myeloid-derived suppressor cells. Cancer Res.

[139] Alvisi G, Brummelman J, Puccio S, Mazza EM, Tomada EP, Losurdo A, Zanon V, Peano C, Colombo FS, Scarpa A, Alloisio M, Vasanthakumar A, Roychoudhuri R (2020). IRF4 instructs effector Treg differentiation and immune suppression in human cancer. J Clin Invest.

[140] Seo H, Gonzalez-Avalos E, Zhang W, Ramchandani P, Yang C, Lio CJ, Rao A, Hogan PG (2021). BATF and IRF4 cooperate to counter exhaustion in tumor-infiltrating CAR T cells. Nat Immunol.

[141] Suthen S, Lim CJ, Nguyen PHD, Dutertre CA, Lai HLH, Wasser M, Chua C, Lim TKH, Leow WQ, Loh TJ, Wan WK, Pang YH, Soon G (2022). Hypoxia-driven immunosuppression by Treg and type-2 conventional dendritic cells in HCC. Hepatology.

[142] Marrugal A, Ojeda L, Paz-Ares L, Molina-Pinelo S, Ferrer I (2016). Proteomic-Based Approaches for the Study of Cytokines in Lung Cancer. Dis Markers.

[143] Dhainaut M, Rose SA, Akturk G, Wroblewska A, Nielsen SR, Park ES, Buckup M, Roudko V, Pia L, Sweeney R, Le Berichel J, Wilk CM, Bektesevic A (2022). Spatial CRISPR genomics identifies regulators of the tumor microenvironment. Cell.

[144] Tang N, Cheng C, Zhang X, Qiao M, Li N, Mu W, Wei XF, Han W, Wang H. TGF-beta inhibition via CRISPR promotes the long-term efficacy of CAR T cells against solid tumors. JCI Insight. 2020;5(4):e133977. 10.1172/jci.insight.133977 PMC7101140 31999649

[145] Kloss CC, Lee J, Zhang A, Chen F, Melenhorst JJ, Lacey SF, Maus MV, Fraietta JA, Zhao Y, June CH (2018). Dominant-Negative TGF-beta Receptor Enhances PSMA-Targeted Human CAR T Cell Proliferation And Augments Prostate Cancer Eradication. Mol Ther.

[146] Hou AJ, Chang ZL, Lorenzini MH, Zah E, Chen YY (2018). TGF-beta-responsive CAR-T cells promote anti-tumor immune function. Bioeng Transl Med.

[147] Mannino MH, Zhu Z, Xiao H, Bai Q, Wakefield MR, Fang Y (2015). The paradoxical role of IL-10 in immunity and cancer. Cancer Lett.

[148] Sullivan KM, Jiang X, Guha P, Lausted C, Carter JA, Hsu C, Labadie KP, Kohli K, Kenerson HL, Daniel SK, Yan X, Meng C, Abbasi A (2023). Blockade of interleukin 10 potentiates antitumour immune function in human colorectal cancer liver metastases. Gut.

[149] Batchu RB, Gruzdyn OV, Mahmud EM, Chukr F, Dachepalli R, Manmari SK, Mostafa G, Weaver DW, Gruber SA (2018). Inhibition of Interleukin-10 in the tumor microenvironment can restore mesothelin chimeric antigen receptor T cell activity in pancreatic cancer in vitro. Surgery.

[150] Avanzi MP, Yeku O, Li X, Wijewarnasuriya DP, van Leeuwen DG, Cheung K, Park H, Purdon TJ, Daniyan AF, Spitzer MH, Brentjens RJ (2018). Engineered Tumor-Targeted T Cells Mediate Enhanced Anti-Tumor Efficacy Both Directly and through Activation of the Endogenous Immune System. Cell Rep.

[151] Valkenburg KC, de Groot AE, Pienta KJ (2018). Targeting the tumour stroma to improve cancer therapy. Nat Rev Clin Oncol.

[152] Wu Z, Sweet RA, Hoyne GF, Simeonovic CJ, Parish CR. Acute T-Cell-Driven Inflammation Requires the Endoglycosidase Heparanase-1 from Multiple Cell Types. Int J Mol Sci. 2022;23(9):4625. 10.3390/ijms23094625 PMC9105945 35563015

[153] Caruana I, Savoldo B, Hoyos V, Weber G, Liu H, Kim ES, Ittmann MM, Marchetti D, Dotti G (2015). Heparanase promotes tumor infiltration and antitumor activity of CAR-redirected T lymphocytes. Nat Med.

[154] Huang Y, Kim BYS, Chan CK, Hahn SM, Weissman IL, Jiang W (2018). Improving immune-vascular crosstalk for cancer immunotherapy. Nat Rev Immunol.

[155] Ma W, Wang Y, Zhang R, Yang F, Zhang D, Huang M, Zhang L, Dorsey JF, Binder ZA, O'Rourke DM, Fraietta JA, Gong Y, Fan Y (2021). Targeting PAK4 to reprogram the vascular microenvironment and improve CAR-T immunotherapy for glioblastoma. Nat Cancer.

[156] Zhang D, Li AM, Hu G, Huang M, Yang F, Zhang L, Wellen KE, Xu X, Conn CS, Zou W, Kahn M, Rhoades SD, Weljie AM (2023). PHGDH-mediated endothelial metabolism drives glioblastoma resistance to chimeric antigen receptor T cell immunotherapy. Cell Metab.

[157] Bule P, Aguiar SI, Aires-Da-Silva F, Dias JNR. Chemokine-Directed Tumor Microenvironment Modulation in Cancer Immunotherapy. Int J Mol Sci. 2021;22(18):9804. 10.3390/ijms22189804 PMC8464715 34575965

[158] Xu N, Palmer DC, Robeson AC, Shou P, Bommiasamy H, Laurie SJ, Willis C, Dotti G, Vincent BG, Restifo NP, Serody JS. STING agonist promotes CAR T cell trafficking and persistence in breast cancer. J Exp Med. 2021;218(2):e20200844. 10.1084/jem.20200844 PMC7780733 33382402

[159] Dimitri A, Herbst F, Fraietta JA (2022). Engineering the next-generation of CAR T-cells with CRISPR-Cas9 gene editing. Mol Cancer.

[160] Huang Y, Jia A, Wang Y, Liu G (2023). CD8(+) T cell exhaustion in anti-tumour immunity: The new insights for cancer immunotherapy. Immunology.

[161] Wherry EJ, Ha SJ, Kaech SM, Haining WN, Sarkar S, Kalia V, Subramaniam S, Blattman JN, Barber DL, Ahmed R (2007). Molecular signature of CD8+ T cell exhaustion during chronic viral infection. Immunity.

[162] Sen DR, Kaminski J, Barnitz RA, Kurachi M, Gerdemann U, Yates KB, Tsao HW, Godec J, LaFleur MW, Brown FD, Tonnerre P, Chung RT, Tully DC (2016). The epigenetic landscape of T cell exhaustion. Science.

[163] McLane LM, Abdel-Hakeem MS, Wherry EJ (2019). CD8 T Cell Exhaustion During Chronic Viral Infection and Cancer. Annu Rev Immunol.

[164] Pritykin Y, van der Veeken J, Pine AR, Zhong Y, Sahin M, Mazutis L, Pe'er D, Rudensky AY, Leslie CS (2021). A unified atlas of CD8 T cell dysfunctional states in cancer and infection. Mol Cell.

[165] Satpathy AT, Granja JM, Yost KE, Qi Y, Meschi F, McDermott GP, Olsen BN, Mumbach MR, Pierce SE, Corces MR, Shah P, Bell JC, Jhutty D (2019). Massively parallel single-cell chromatin landscapes of human immune cell development and intratumoral T cell exhaustion. Nat Biotechnol.

[166] Bengsch B, Ohtani T, Khan O, Setty M, Manne S, O'Brien S, Gherardini PF, Herati RS, Huang AC, Chang KM, Newell EW, Bovenschen N, Pe'er D (2018). Epigenomic-Guided Mass Cytometry Profiling Reveals Disease-Specific Features of Exhausted CD8 T Cells. Immunity.

[167] Khan O, Giles JR, McDonald S, Manne S, Ngiow SF, Patel KP, Werner MT, Huang AC, Alexander KA, Wu JE, Attanasio J, Yan P, George SM (2019). TOX transcriptionally and epigenetically programs CD8(+) T cell exhaustion. Nature.

[168] Chen J, Lopez-Moyado IF, Seo H, Lio CJ, Hempleman LJ, Sekiya T, Yoshimura A, Scott-Browne JP, Rao A (2019). NR4A transcription factors limit CAR T cell function in solid tumours. Nature.

[169] Seo H, Chen J, Gonzalez-Avalos E, Samaniego-Castruita D, Das A, Wang YH, Lopez-Moyado IF, Georges RO, Zhang W, Onodera A, Wu CJ, Lu LF, Hogan PG (2019). TOX and TOX2 transcription factors cooperate with NR4A transcription factors to impose CD8(+) T cell exhaustion. Proc Natl Acad Sci U S A.

[170] LaFleur MW, Nguyen TH, Coxe MA, Miller BC, Yates KB, Gillis JE, Sen DR, Gaudiano EF, Al Abosy R, Freeman GJ, Haining WN, Sharpe AH (2019). PTPN2 regulates the generation of exhausted CD8(+) T cell subpopulations and restrains tumor immunity. Nat Immunol.

[171] Chen Z, Ji Z, Ngiow SF, Manne S, Cai Z, Huang AC, Johnson J, Staupe RP, Bengsch B, Xu C, Yu S, Kurachi M, Herati RS (2019). TCF-1-Centered Transcriptional Network Drives an Effector versus Exhausted CD8 T Cell-Fate Decision. Immunity.

[172] Siddiqui I, Schaeuble K, Chennupati V, Fuertes Marraco SA, Calderon-Copete S, Pais Ferreira D, Carmona SJ, Scarpellino L, Gfeller D, Pradervand S, Luther SA, Speiser DE, Held W (2019). Intratumoral Tcf1(+)PD-1(+)CD8(+) T Cells with Stem-like Properties Promote Tumor Control in Response to Vaccination and Checkpoint Blockade Immunotherapy. Immunity.

[173] Wiede F, Lu KH, Du X, Liang S, Hochheiser K, Dodd GT, Goh PK, Kearney C, Meyran D, Beavis PA, Henderson MA, Park SL, Waithman J (2020). PTPN2 phosphatase deletion in T cells promotes anti-tumour immunity and CAR T-cell efficacy in solid tumours. EMBO J.

[174] Lynn RC, Weber EW, Sotillo E, Gennert D, Xu P, Good Z, Anbunathan H, Lattin J, Jones R, Tieu V, Nagaraja S, Granja J, de Bourcy CFA (2019). c-Jun overexpression in CAR T cells induces exhaustion resistance. Nature.

[175] Pardoll DM (2012). The blockade of immune checkpoints in cancer immunotherapy. Nat Rev Cancer.

[176] Dolina JS, Van Braeckel-Budimir N, Thomas GD, Salek-Ardakani S (2021). CD8(+) T Cell Exhaustion in Cancer. Front Immunol.

[177] Cherkassky L, Morello A, Villena-Vargas J, Feng Y, Dimitrov DS, Jones DR, Sadelain M, Adusumilli PS (2016). Human CAR T cells with cell-intrinsic PD-1 checkpoint blockade resist tumor-mediated inhibition. J Clin Invest.

[178] Rafiq S, Yeku OO, Jackson HJ, Purdon TJ, van Leeuwen DG, Drakes DJ, Song M, Miele MM, Li Z, Wang P, Yan S, Xiang J, Ma X (2018). Targeted delivery of a PD-1-blocking scFv by CAR-T cells enhances anti-tumor efficacy in vivo. Nat Biotechnol.

[179] Heindl A, Khan AM, Rodrigues DN, Eason K, Sadanandam A, Orbegoso C, Punta M, Sottoriva A, Lise S, Banerjee S, Yuan Y (2018). Microenvironmental niche divergence shapes BRCA1-dysregulated ovarian cancer morphological plasticity. Nat Commun.

[180] Li Y, Wu X, Fang D, Luo Y (2024). Informing immunotherapy with multi-omics driven machine learning. NPJ Digit Med.

[181] Xu H, Clemenceau JR, Park S, Choi J, Lee SH, Hwang TH (2022). Spatial heterogeneity and organization of tumor mutation burden with immune infiltrates within tumors based on whole slide images correlated with patient survival in bladder cancer. J Pathol Inform.

[182] Gordon KS, Kyung T, Perez CR, Holec PV, Ramos A, Zhang AQ, Agarwal Y, Liu Y, Koch CE, Starchenko A, Joughin BA, Lauffenburger DA, Irvine DJ (2022). Screening for CD19-specific chimaeric antigen receptors with enhanced signalling via a barcoded library of intracellular domains. Nat Biomed Eng.

[183] Goodman DB, Azimi CS, Kearns K, Talbot A, Garakani K, Garcia J, Patel N, Hwang B, Lee D, Park E, Vykunta VS, Shy BR, Ye CJ (2022). Pooled screening of CAR T cells identifies diverse immune signaling domains for next-generation immunotherapies. Sci Transl Med.

[184] Daniels KG, Wang S, Simic MS, Bhargava HK, Capponi S, Tonai Y, Yu W, Bianco S, Lim WA (2022). oding CAR T cell phenotype using combinatorial signaling motif libraries and machine learning. Science.

[185] Bulik-Sullivan B, Busby J, Palmer CD, Davis MJ, Murphy T, Clark A, Busby M, Duke F, Yang A, Young L, Ojo NC, Caldwell K, Abhyankar J, et al. Deep learning using tumor HLA peptide mass spectrometry datasets improves neoantigen identification. Nat Biotechnol. 2018;37:55–63. 10.1038/nbt.4313 30556813

[186] Andreatta M, Nielsen M (2016). Gapped sequence alignment using artificial neural networks: application to the MHC class I system. Bioinformatics.

[187] Rammensee H, Bachmann J, Emmerich NP, Bachor OA, Stevanović S (1999). SYFPEITHI: database for MHC ligands and peptide motifs. Immunogenetics.

[188] Racle J, Michaux J, Rockinger GA, Arnaud M, Bobisse S, Chong C, Guillaume P, Coukos G, Harari A, Jandus C, Bassani-Sternberg M, Gfeller D (2019). Robust prediction of HLA class II epitopes by deep motif deconvolution of immunopeptidomes. Nat Biotechnol.

[189] Stražar M, Park J, Abelin JG, Taylor HB, Pedersen TK, Plichta DR, Brown EM, Eraslan B, Hung YM, Ortiz K, Clauser KR, Carr SA, Xavier RJ (2023). HLA-II immunopeptidome profiling and deep learning reveal features of antigenicity to inform antigen discovery. Immunity.

[190] De Mattos-Arruda L, Vazquez M, Finotello F, Lepore R, Porta E, Hundal J, Amengual-Rigo P, Ng CKY, Valencia A, Carrillo J, Chan TA, Guallar V, McGranahan N (2020). Neoantigen prediction and computational perspectives towards clinical benefit: recommendations from the ESMO Precision Medicine Working Group. Ann Oncol.

[191] Wu J, Wang W, Zhang J, Zhou B, Zhao W, Su Z, Gu X, Wu J, Zhou Z, Chen S (2019). DeepHLApan: A Deep Learning Approach for Neoantigen Prediction Considering Both HLA-Peptide Binding and Immunogenicity. Front Immunol.

[192] Zhang Y, Li Y, Li T, Shen X, Zhu T, Tao Y, Li X, Wang D, Ma Q, Hu Z, Liu J, Ruan J, Cai J (2019). Genetic Load and Potential Mutational Meltdown in Cancer Cell Populations. Mol Biol Evol.

[193] Greenman C, Stephens P, Smith R, Dalgliesh GL, Hunter C, Bignell G, Davies H, Teague J, Butler A, Stevens C, Edkins S, O'Meara S, Vastrik I (2007). Patterns of somatic mutation in human cancer genomes. Nature.

[194] Zeng Z, Gu SS, Wong CJ, Yang L, Ouardaoui N, Li D, Zhang W, Brown M, Liu XS (2022). Machine learning on syngeneic mouse tumor profiles to model clinical immunotherapy response. Sci Adv.

[195] Lu Z, Chen H, Jiao X, Zhou W, Han W, Li S, Liu C, Gong J, Li J, Zhang X, Wang X, Peng Z, Qi C, et al. Prediction of immune checkpoint inhibition with immune oncology-related gene expression in gastrointestinal cancer using a machine learning classifier. J Immunother Cancer. 2020;8(2):e000631. 10.1136/jitc-2020-000631 PMC7430448 32792359

[196] Jiang Y, Zhang Z, Wang W, Huang W, Chen C, Xi S, Ahmad MU, Ren Y, Sang S, Xie J, Wang JY, Xiong W, Li T (2023). Biology-guided deep learning predicts prognosis and cancer immunotherapy response. Nat Commun.

[197] Addala V, Newell F, Pearson JV, Redwood A, Robinson BW, Creaney J, Waddell N (2024). Computational immunogenomic approaches to predict response to cancer immunotherapies. Nat Rev Clin Oncol.

[198] Zhang Z, Wang ZX, Chen YX, Wu HX, Yin L, Zhao Q, Luo HY, Zeng ZL, Qiu MZ, Xu RH (2022). Integrated analysis of single-cell and bulk RNA sequencing data reveals a pan-cancer stemness signature predicting immunotherapy response. Genome Med.

[199] Gohil SH, Iorgulescu JB, Braun DA, Keskin DB, Livak KJ (2021). Applying high-dimensional single-cell technologies to the analysis of cancer immunotherapy. Nat Rev Clin Oncol.

